# From Survey Results to a Decision-Making Matrix for Strategic Planning in Healthcare: The Case of Clinical Pathways

**DOI:** 10.3390/ijerph19137806

**Published:** 2022-06-25

**Authors:** Lavinia Bianco, Salvatore Raffa, Paolo Fornelli, Rita Mancini, Angela Gabriele, Francesco Medici, Claudia Battista, Stefania Greco, Giuseppe Croce, Aldo Germani, Simona Petrucci, Paolo Anibaldi, Valerio Bianco, Mario Ronchetti, Giorgio Banchieri, Christian Napoli, Maria Piane

**Affiliations:** 1 Department of Public Health and Infectious Diseases, “Sapienza” University of Rome, Piazzale Aldo Moro 5, 00185 Rome, Italy; lavinia.bianco@uniroma1.it; 2 Department of Clinical and Molecular Medicine, “Sapienza” University of Rome, Viale Regina Elena 291, 00161 Rome, Italy; salvatore.raffa@uniroma1.it (S.R.); rita.mancini@uniroma1.it (R.M.); aldo.germani@uniroma1.it (A.G.); simona.petrucci@uniroma1.it (S.P.); 3 Sant’Andrea University Hospital, Via di Grottarossa 1035-1039, 00189 Rome, Italy; panibaldi@ospedalesantandrea.it (P.A.); christian.napoli@uniroma1.it (C.N.); 4 Associazione Italiana per la Qualità della Assistenza Sanitaria e Sociale (ASIQUAS), Largo Konrad Adenauer 1/B, 00144 Rome, Italy; fornellip26@gmail.com (P.F.); ronchetti.mario@gmail.com (M.R.); giorgio.banchieri@gmail.com (G.B.); 5 Department of Social Sciences and Economics, “Sapienza” University of Rome, Via Salaria 113, 00198 Rome, Italy; 6 Azienda Sanitaria Locale (ASL) Frosinone District C and D, Via De Bosis-03043 Cassino, Via Piemonte, 03039 Sora, Italy; angela.gabriele1@virgilio.it; 7 Azienda Ospedaliera San Camillo-Forlanini, Circonvallazione Gianicolense, 87, 00152 Rome, Italy; fam.medici@mac.com (F.M.); stefaniagreco@libero.it (S.G.); 8 Department of Mental Health and Pathological Addictions, Azienda Sanitaria Locale (ASL) RM 6, Via Borgo Garibaldi 12, 00041 Albano Laziale, Italy; claudia.battista@gmail.com; 9 Internal Medicine Unit, “G. Mazzini” Hospital, ASL 4 Teramo, Piazza Italia 1, 64100 Teramo, Italy; gpp.crc@me.com; 10 Department of Medical Surgical Sciences and Translational Medicine, “Sapienza” University of Rome, Via di Grottarossa 1035/1039, 00189 Rome, Italy; valeriobianco99@gmail.com

**Keywords:** clinical pathways, comparison tool, strategic planning, COVID-19, healthcare system

## Abstract

Background: It is a well-known fact that the information obtained from a survey can be used in a healthcare organizational analysis; however, it is very difficult to compare the different results found in the literature to each other, even through the use of metanalysis, as the methodology is often not consistent. Methods: Data from a survey analyzing the organizational and managerial responses adopted in pathology-specific clinical pathways (CPs) during the first two waves of the COVID-19 pandemic were used for constructing a decisional matrix, a tool called SPRIS system, consisting of four different sheets. The first sheet reports the results of the survey and, using a streetlight color system, identifies strengths and weaknesses; the second one, by assigning a priority score, establishes the priority of intervention on each of the strengths and weaknesses identified; the third sheet reports the subjective items of the questionnaire in order to identify threats and opportunities and their probability of happening; in the last sheet, a SWOT Analysis is used to calculate the performance index of the whole organization. Results: The SPRIS system, applied to data concerning the adaptation of four CPs to the COVID-19 pandemic, showed that, whereas all the CPs had a good performance index, some concerns remained unsolved and need be addressed. Conclusions: The SPRIS system showed to be an easily constructed tool that is able to give an overview of the organization analyzed by the survey and to produce an index that can be used in a direct quality comparison between different services or organizations.

## 1. Introduction

In face of the growing importance of evidence-based interventions (EBIs), based on the concept that decisions and interventions must use the most appropriate information and evidence [1,2], there has been an increasing trend regarding the use of quality measurement of healthcare systems [3,4]. However, in contrast to evidence-based medicine (EBM), obtaining evidence for public health policy and its quality is much more complex, as the policy process involves a series of steps whose evidence is complex to acquire. In fact, the effectiveness of interventions, feasibility of the organization, and implementation, which are less commonly covered by research evidence, are often difficult to decipher, susceptible to interpretations, and apt to be misinterpreted [5,6].

It is suggested that the best way to obtain evidence about a policy that has already been introduced is through interviews or surveys specifically designed to measure the quality of care and the policy [3,6,7]. In fact, a policy is largely a trial-and-error process, and, therefore, the scientific community can provide a crucial contribution by providing rigorous and fast evaluations of it [6]. Moreover, survey research is an important methodology, and it is considered to be the easiest way of collecting considerable information from which one can draw a meaningful conclusion in a relatively short period, sometimes with a direct economic impact [6,7,8,9,10].

However, interviews and survey often lack objectivity and leave too much freedom in the interpretations of the results. Even if it is believed that the intellectual rigor of EBM is applicable in this context [6], the lack of adequate data may threaten the validity of results, as complete and transparent reporting is necessary for readers to adequately assess the biases, strengths, and weaknesses of the study and the generalizability of the results [8]. In fact, if the results cannot be generalized, it can be very hard to use them as evidence when creating a new public health policy [4]. This is something that the scientific community has already highlighted as a problem, especially for patient-reported outcomes (PROs), often trying to solve it with the standardization of the scores from different instruments as standardized response means (SRMs); however, since standard deviations (SD) may vary substantially from one study to another one, treatment effects that are homogeneous when expressed in their original unit can become heterogeneous when expressed as SRMs [11].

It has been suggested, as a possible solution, to create a registry to collect all the survey research and also to regulate what sort of analysis will be carried out [10]. However, at the moment, the comparison between surveys is often made by using only the items in common; on the contrary, for the other items or for indicators based on the authors’ opinion, the comparability cannot be achieved, and, therefore, it is omitted, determining the loss of any comparison [12].

Therefore, it is evident that finding a common data analysis method is a priority [10], especially to enhance the comparison across countries and over time [13]. Thus, the qualitative responses obtained from a survey should be transformed into measurable values that are able to identify and weight the strengths and weaknesses that emerge, highlighting critical issues of the organization and establishing intervention priorities to reach an adequate level of medical assistance.

### Aim of the Current Study

In this scientific context, we propose a new tool, the Streetlight PRIority Swot system (SPRIS), in order to evaluate the quality of health services and to suggest improvement actions emerged by a survey. The SPRIS was subsequently applied to the results of a previously performed survey that aimed to analyze the organizational and managerial responses adopted in four pathology-specific clinical pathway (CPs) during the COVID-19 pandemic [14]. This methodology may represent a proposal model for measuring the quality of an activity (or a service) included into public health policies.

## 2. Materials and Methods

The COVID survey, used as starting point to be evaluated [14], consists of 37 items grouped by thematic area into eight sections. The Questionnaire Sections are as follows:

1. Context analysis;
2. Patients’ access to Care Pathways (CPs)/Operational Units (OUs);
3. Impact on the treatment of non-COVID patients in the CPs;
4. Impact on the treatment of patients also SARS-CoV-2 infected in the CPs;
5. Impact of the COVID-19 pandemic on patient management;
6. Structural and organizational changes of the CPs/OUs;
7. Procedures and recommendations for healthcare professionals/users;
8. Training, information, and management of health workers in the pandemic era.

The investigated OUs belong to four different Local Health Units (ASL)/Hospitals (AO) and are divided as follows: seven OUs to the hereditary breast–ovarian cancers CP (inserted as CP1 in the SPRIS), six OUs to the autism spectrum disorders (DSA) CP (inserted as CP2), six OUs to the diabetes CP (inserted as CP3), and five OUs related to the heart failure CP (inserted as CP4) [14].

During the previous survey study, the means and standard deviations (SDs) of a bipolar 4-point Likert scales were calculated for each question of the survey (“yes” is equal to 4, “enough” to 3, “not enough” to 2, “not at all” to 1, and “not applicable” to 0) [15]. A mean score ≥1.80 was considered to be the cutoff for an acceptable level of performance of the CP, and a mean score ≥2.99 was the cutoff for a good level of performance of the CP; a mean score <1.80 was considered as a not acceptable level of performance [14]. The methodology adopted in the survey will not be reported in detail in this paper, but it can be found in the previous article [14] and in Supplementary Material S2. In regard to the Likert scale, this is a very common method of attitude measurement in which the respondent is asked to check one of five possible answers, each one associated with a score from 0 to 4; the final results are the sum of the point values for the choices selected [15].

In the present study, the authors decided to go one step further, performing and testing the SPRIS, which includes four Microsoft Excel sheets.

This tool works as a strategic management process tool; it allows an immediate and clear view of critical areas, gives a priority score to each found criticism, and calculates a performance index though a Next-Generation SWOT Analysis, as a measure of the quality of the activity/service addressed. The tool allows a depth of data reading according to different degrees of aggregation, and it provides an answer (feedback) which can be used to evaluate the organizational performance.

In the **first sheet** (*Streetlight color system* sheet) are inserted the results of the previously cited survey [14], showing the results of each analyzed item and allowing users to recognize all the items that represented strengths (colored in green) and weaknesses (colored in yellow and red).

In the **second sheet** (*Priority scores* sheet), the mean scores of the items are classified with a graduated scale from 1 to 10, with the intention to establish how important those items are for the strategic planning and to establish the priority of each improving action.

The survey used [14] reports only objective items, those that do not investigate opportunities and threats external to the organization, whose priority scores can be used directly by themselves. In the case of surveys including subjective items, those being externa, that have a probability to happen, a **third sheet** of the tool (*Delphi-like* sheet) is foreseen to analyze the probabilities that the opportunities and threats will occur. The probability is calculated with the Delphi-like method, which extrapolates for each opportunity and threat a median of the single opinions (expressed as a percentage that the event will occur) given by a team of experts; if a median cannot be calculated, the experts, after the necessary comparisons, reach a shared opinion. Since the survey that is used in this article has no subjective items [14], as there are no items regarding elements external to the CPs’ inner workings, it was not necessary to apply the *Delphi-like* sheet. This does not affect the analysis of this survey, as the *Delphi-like* sheet is an extra-analysis-tool, valid only for surveys reporting subjective items, and it can, therefore, be excluded from the analysis of surveys that lack such items. Overall, it was developed as a further support, and it can be found in Supplementary Materials S1.

Lastly, in the **fourth sheet** (*NGSA* sheet), strengths and weaknesses are inserted into a new type of SWOT Analysis, the Next-Generation SWOT Analysis (NGSWOT Analysis or NGSA). The classic SWOT shows an evident weakness: the four groups of elements are inserted in a descriptive way, and it is not possible to understand neither if a favorable or unfavorable picture for the implementation of a goal is produced, nor where it is necessary to take improving actions. Therefore, the NGSWOT Analysis was designed to reduce this criticism, as the insertion of the numerical values obtained for the strengths and weaknesses in SWOT allows users to correlate all data as a whole and calculate an indicator of the organization’s performance, the performance index.

**Figure 1:**
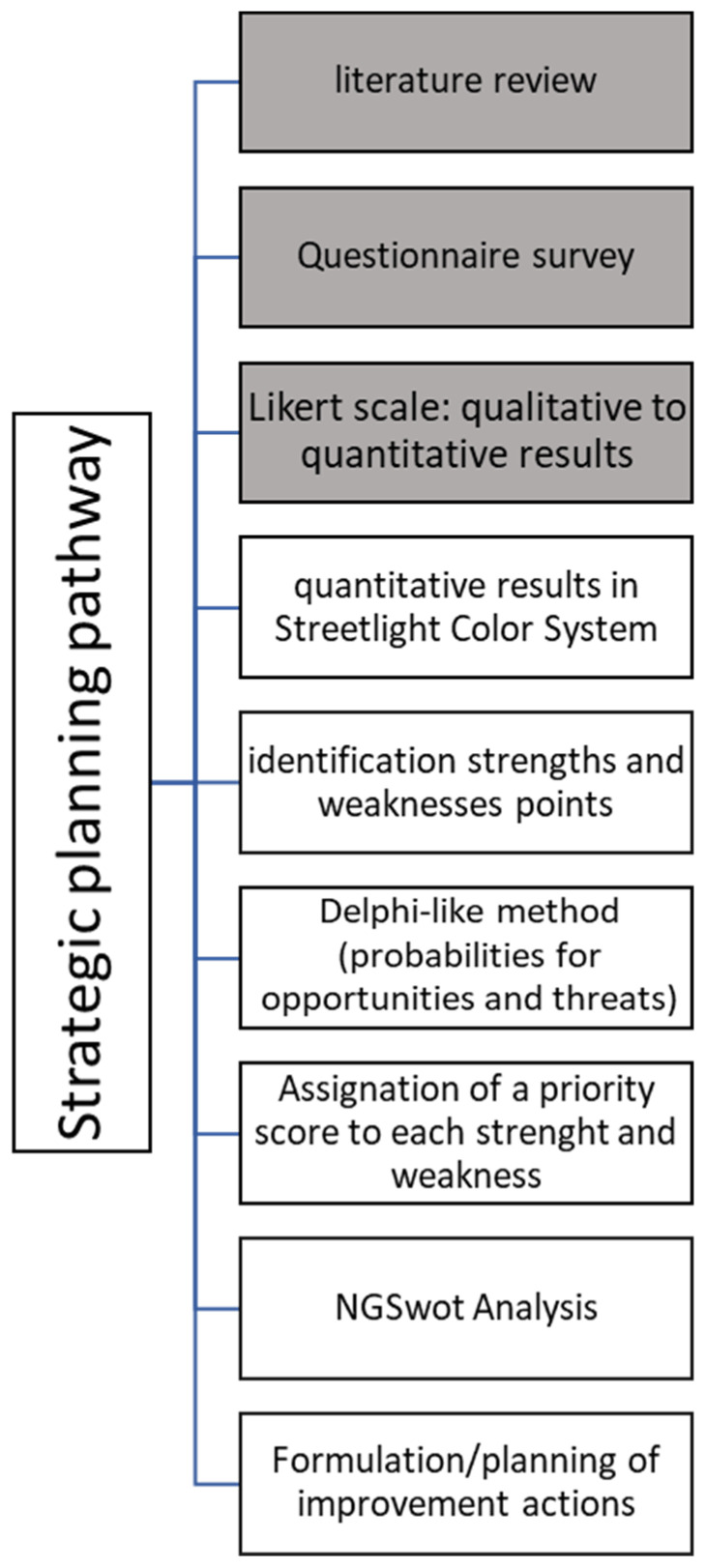
Workflow chart. The gray squares indicate the methodology of the first study published by the authors [14].

**Figure 2:**
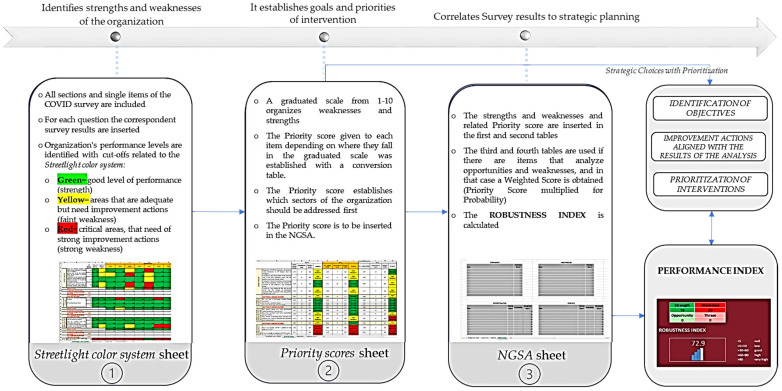
Graphic representation of the construction of the Excel sheets of SPRIS and the meaning of each one of them.

The whole process is summarized in Figure 1, while the construction of the sheets is shown in Figure 2 and in Supplementary Materials S1.

### 2.1. First Sheet: Streetlight Color System

The objective of this first sheet is to photograph a complete picture, by successive levels of aggregation, of the reality that is being investigated. The tool is able to evaluate the parameters entered and identify, both horizontally (at the parameter level) and vertically (at the organizational level), the most critical areas of the organization/department/operational units being analyzed.

**Figure 3:**
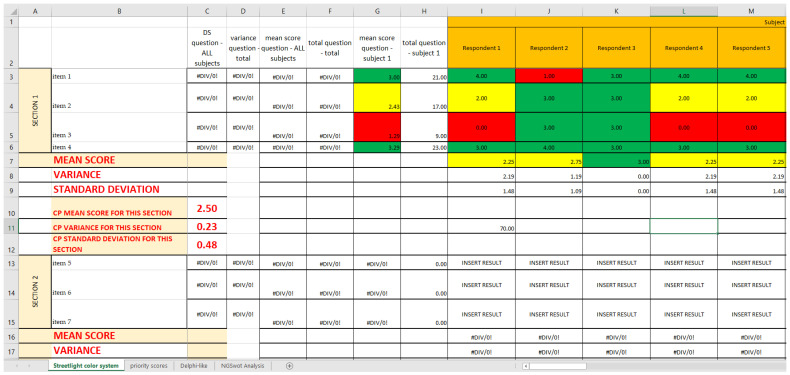
Example of SPRIS application. Photograph of the complete picture given by the *Streetlight color system* sheet, showing the first three Questionnaire Sections of the questionnaire for two CPs.

Using the already established cutoffs [14], we created a graphic model with three different colors, namely green, yellow, and red, a so-called “streetlight color system”, thanks to Excel Conditional Formatting; it allows a synoptic and immediate reading of the results. The three colors highlight the structures on which an improvement intervention is necessary. In this way, it is clear from just a glance that the areas that already have a good level of performance are colored in green; the ones that are adequate but can be modified to achieve better results are colored in yellow; and, lastly, the ones that need immediate corrections are colored in red (critical areas) (Figure 3). Since both red and yellow have issues that need to be addressed, we classified them as strong and faint weaknesses, respectively, while green was classified as strength.

### 2.2. Second Sheet: Priority Scores

The streetlight color system only highlights strengths and weaknesses but does not organize them and does not give them a priority, which is the feature needed when constructing a decisional matrix. Therefore, once that the streetlight color system was created, it was deemed necessary to give each of the found weakness and strength a priority of intervention through a classification scale. In order to fulfill this sheet, a panel of experts is necessary. The panel must be composed by at least three components: one expert in healthcare services evaluation, one expert in public health, and one expert in healthcare services management. The items with the lowest scores are the ones that have to be addressed first in the decision matrix.

The actions to improve the quality of an analyzed service can be organized with a timing that depends on the priority obtained (priority score) from the classification scale.

#### 2.2.1. Classification Scale

The cutoffs for each group of the classification scale are generate by dividing the main interval into ten sub-intervals of similar “length” that are then classified in a scale from 1 to 10, with weaknesses going from 1 to 7 and strengths from 8 to 10 (Table 1). In our case, the length of each sub-interval is about 0.4, as the questionnaire we bring as an example has a scoring system from 0 to 4, but the system can be applied to any range. For instance, if the questionnaire had a scoring system from 0 to 20, the 10 sub-intervals would be about 2 points each, and the three main intervals about 6.7 points each.

To summarize, the main cutoffs allow the synoptic reading of the *Streetlight color system* sheet, while the sub-intervals allow to give the priority score.

#### 2.2.2. Conversion Scale

To insert in the NGSWOT Analysis the correct “weight” for each item, we established a conversion scale, so that, for each cutoff, there is a corresponding score that is different for strong weaknesses, faint weaknesses, and strengths. This is necessary, as the classification scale by itself gives the order of importance but not a score that can be inserted into a SWOT Analysis.

Weaknesses have a descending consecutive scoring system (as the items with the lowest classification scale number are more “important”): from 5 to 2 for strong weaknesses, and from 1.5 to 0.5 for faint weaknesses. In order to differentiate between strong and faint weaknesses, the former would have a scoring system by one point and the latter by half a point.

The strengths, instead, have an increasing consecutive scoring system by 1.5 points, from 1.5 to 4.5; it is used a system by 1.5 points to compensate for the fact that the value of all the weaknesses, especially the strong weaknesses, should be balanced by the strengths found in the same setting, while still highlighting the critical areas. Therefore, the strength with the highest priority score should be about 0.5 points lower than the strong weakness with the highest priority score; at the same time, the strength with the lowest priority score should be able to compensate for only the faint weaknesses and nothing higher. In other words, the classification n.8 had to have a priority score of 1.5 (equal to classification 5), and the classification n.10 had to have a priority score of 4.5 (0.5 lower than classification 1); from there, the 1.5 system was an obvious conclusion (Table 2).

**Figure 4:**
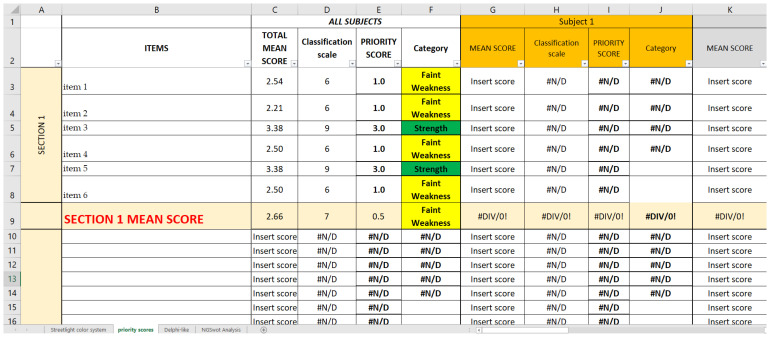
Example of *Priority scores* sheet of SPRIS; for each mean score, we report the correspondent classification scale, the priority score, and the category, following the conversion table. The construction of this second Excel sheet depends on the level of depth of the organizational analysis that is being applied. The analysis can be carried out at different levels: macro-dimensional, if one chooses to insert the data (mean scores) concerning the CP as a whole, with both horizontal and vertical reading; or micro-dimensional, if one choses to insert the mean scores for individual items, using only the horizontal or only the vertical.

This priority scores so obtained are reported in the *Priority scores* sheet (Figure 4), alongside the mean score they are referring to and the category they belong to (strength and strong or faint weakness).

### 2.3. Last Sheet: Next-Generation SWOT Analysis

The NGSA calculates a performance index, which is a measure of the quality and of the level of performance that is shown by the activity/service analyzed with the survey, which, in this specific case, is how well—or how badly—the four chosen clinical pathways adjusted to the COVID-19 pandemic.

Based on the value of the calculated index, it can be decided whether it is useful to spend resources into that activity/service. In case of a low performance index, the whole process must be repeated after having acted upon the weaknesses of the chosen activity to have proof that the improvement actions are actually working.

**Figure 5:**
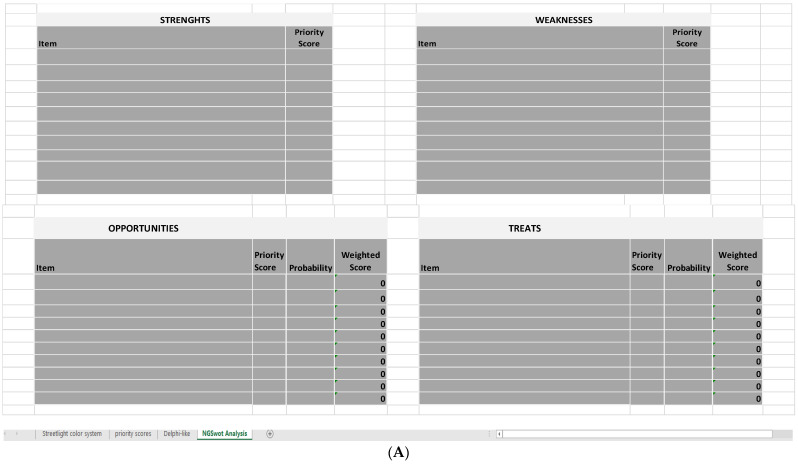

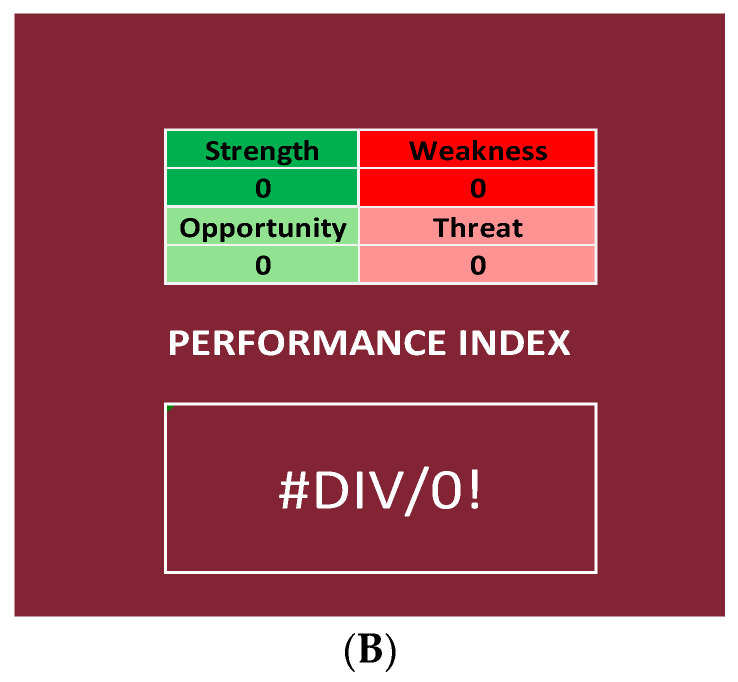
Extraction of the last Excel sheet of SPRIS (*NGSA*). (**A**) Four tables used in the NGSWOT Analysis. (**B**) Sector of the sheet where the performance index is calculated.

The NGSA is constructed as follows: a first table for the strengths and their scores, a second table for the weaknesses (both strong and faint) and their scores, and two more tables for opportunities and threats and their weighted scores (Figure 5A).

The first two tables are compiled by inserting the scores reported in the second Excel sheet (*Priority scores* sheet), depending on the category the item belongs to. The last two tables are compiled by inserting the scores reported in the *Priority scores* sheet and the probabilities reported in the *Delphi-like* sheet; the weighted score is calculated directly by these last two tables, multiplying the priority score of that item for the probability calculated though the Delphi-like method.

As previously stated, in the survey that we bring as an example [14], all the items of the questionnaire are about objective data, intending with this term all those items that do not investigate opportunities and threats external to the organization. In this case, the priority scores can be used as they are, as there is no probability of the event occurring that must be considered. Therefore, it was not necessary to include these last two tables or the process connected to their application.

#### 2.3.1. Performance Index

The NGSA uses the sum of both the priority scores (strength and weakness) and the weighted scores (opportunities and threats) to calculate a performance index, which is a percentage ratio that is calculated by using the following formula:

$$\frac{\left(\mathrm{strength}+\mathrm{opportunity}\right)*100\%}{(\left(\mathrm{strength}+\mathrm{opportunity}\right)+\left(\mathrm{weakness}+\mathrm{threat}\right)}$$

Therefore, the Next-Generation SWOT Analysis differentiates itself from other SWOT Analyses, as it is not only a descriptive method but also an objective analysis system.

In our specific case, it was not necessary to include opportunities and threats in the formula for the performance index:

$$\frac{\mathrm{strength}*100\%}{\left(\mathrm{strength}+\mathrm{weakness}\right)}$$

This index is a measure of the quality of the activity/service analyzed.

#### 2.3.2. Range of the Performance Index

Considering all the results obtained by applying this formula to our data and distributing them in ascending order, it is possible to generate a curve; thanks to the Shapiro–Wilk test, it is possible to state that the performance index is a normally distributed variable, as the *p*-value is higher than 0.05 and, therefore, the distribution of the curves is normal. In fact, it is *p* = 0.46 for the curve created by using the performance indexes regarding the items and *p* = 0.14 for the curve regarding the Questionnaire Sections.

The aim of generating these curves is to have a distribution from which we can identify the range of the percentiles, on which we classify the results:

○ Null if below the tenth percentile;
○ Low if between the tenth percentile and the first quartile;
○ Good if between the first and the second quartile;
○ High if between the second and the third quartile;
○ Very high if between the third and the fourth quartile.

### 2.4. COVID Survey and SPRISS

**Figure 6:**
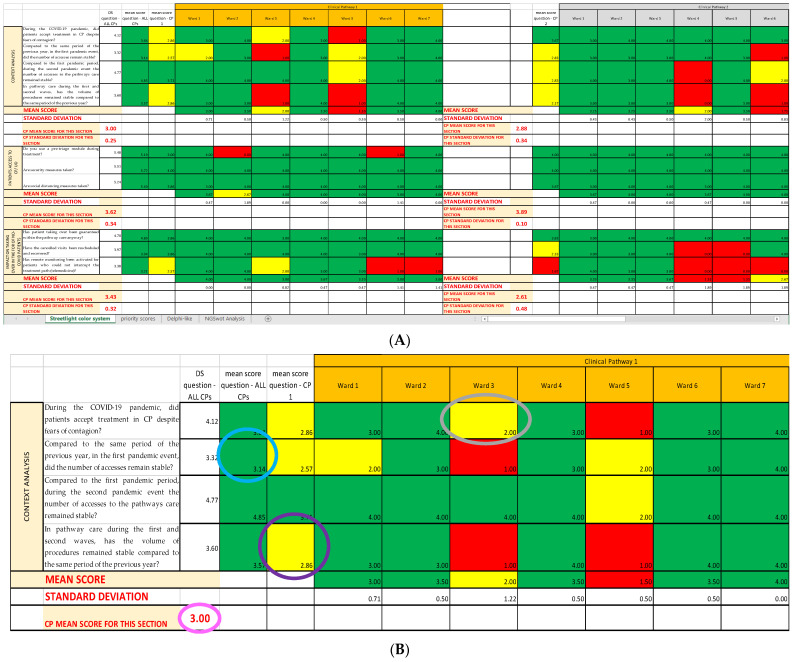

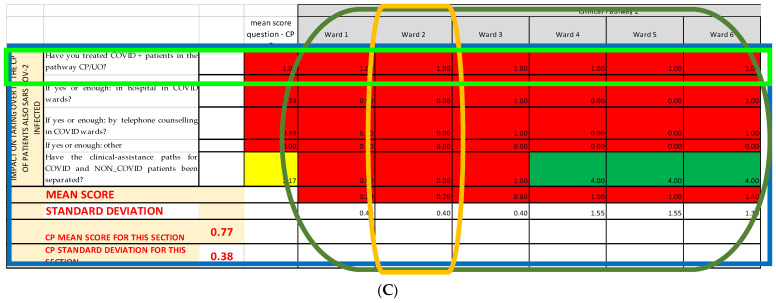
Example of SPRIS (Streetlight PRIority Swot) application. (**A**) Photograph of the complete picture given by the *Streetlight color system* sheet, showing the first three Questionnaire Sections for two of the considered CPs. (**B**) Extraction of the first Questionnaire Section of the first CP. We can read the data about each item/ward (answer score in related cell; the gray circle shows as an example the score of item 1 reached by Ward 3) or about each item/CP (mean score of the answers of a single item for the CP as a whole, shown as an example for item 4 by the purple circle), or about each item/total CPs (mean score of the answers regarding a single item for all the CPs, shown as an example for item 2 by the light blue circle); for each Questionnaire Section of each CP a mean score is calculated (pink circle). (**C**) Extraction of the fourth Questionnaire Section of the second CP. Horizontally, we can find the data about the single question (neon green rectangle) and the fourth Questionnaire Section (blue rectangle); vertically, the data about one specific CP (dark green circle) and one of the first OU/wards (orange rectangle) are reported.

The COVID survey, used as an organizational analysis tool, consists of 37 items grouped by thematic area into eight sections (Questionnaire Sections); all the Questionnaire Sections and the single items of the survey are included in the first Excel sheet (*Streetlight color system* sheet), following the order established in the questionnaire [14]. For each question, we report the Likert scale value correspondent to the qualitative answers given by each operational unit/ward responding to the survey, assembled depending on the CP they belong to (Figure 6A). Thanks to the colors provided with the cutoffs, the reading is both immediate and multidimensional, as it can be read both horizontally and vertically: horizontally, we can find the data about the single question and the Questionnaire Section of the questionnaire; and vertically, the data about each CP and each OU/ward are reported. (Figure 6B).

**Figure 7:**
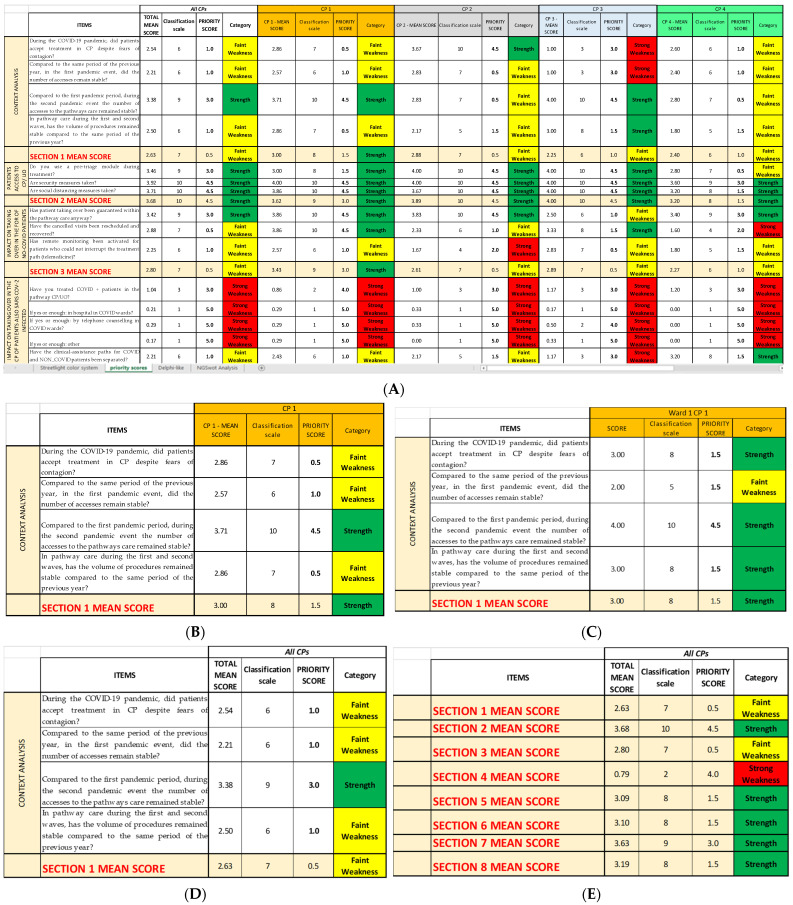
(**A**) Photograph of the *Priority scores* sheet of SPRIS; for each mean score, we report the correspondent classification scale, the priority score, and the category, following the conversion table. (**B**) Extraction of the *Priority scores* sheet regarding the first Questionnaire Section of the survey referring to the first CP considered. (**C**) Extraction regarding the first ward considered (vertical reading of the streetlight color system). (**D**) Extraction regarding the items of the first Questionnaire Section (horizontal reading of the Streetlight color system). (**E**) Extraction regarding all eight Questionnaire Sections (horizontal reading of the streetlight color system).

In the second Excel sheet (*Priority scores* sheet), after having applied the conversion scale (Table 2) to identify the correct priority score of each item of every CP (Figure 7A), it is possible to group and separate the results for each CP (Figure 7B); for each OU/ward (Figure 7C); for each item, regardless of the CP or the OU/ward (Figure 7D); and, lastly, for each Questionnaire Section, regardless not only of the CP or the OU/ward (Figure 7E). The last method might be useful if the analysis must be quickly performed, even if it gives only a superficial overview. In a second moment, for instance, if a Questionnaire Section is categorized as weakness, a more detailed analysis can be added.

**Figure 8:**
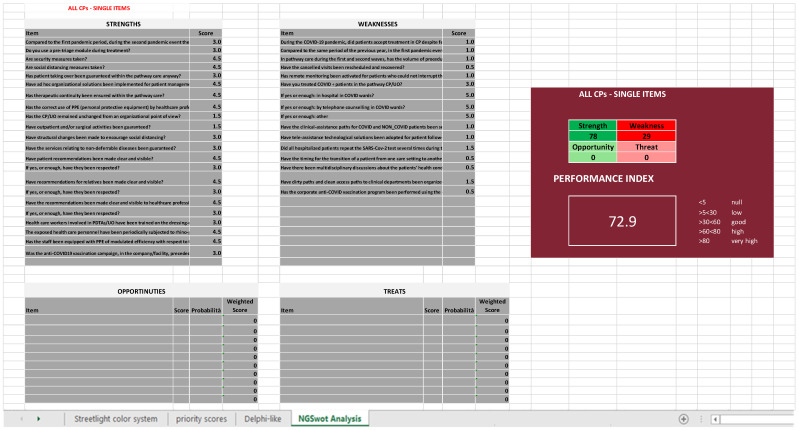
Extraction of the last Excel sheet of SPRIS (*NGSA*). It shows the NGSWOT Analysis compiled by using the priority scores of each item, regardless of the CP or the OU/ward, and the performance index calculated with them.

The results so grouped are then inserted into the last Excel sheet (*NGSA* sheet), constructed with the four tables, as shown in Figure 5A, plus an additional square that reports the total sum of the priority scores for every category (strengths, weaknesses, opportunities, and threats) and the performance index that has been calculated for each chosen depth of analysis (Figure 5B). However, seeing that there are not subjective items, the last two tables have not been filled; therefore, the NGSA sheets all appear similar to the one shown in Figure 8. 

**Figure 9:**
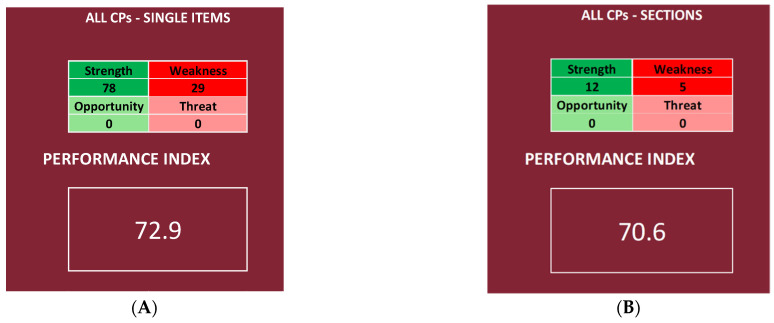
Performance index calculated by using the scores of all four CPs but inserting either the single items (**A**) or only the Questionnaire Sections mean scores (**B**).

It is possible to obtain two different performance indexes, one by inserting in the NGSA the priority scores of the items and the other by using the Questionnaire Sections, regardless of the CP or the OU/ward (Figure 9). The same was performed for each CP, obtaining for each CP two different performance indexes, one by inserting in the NGSA the priority scores of the items (deeper analysis) and the other by using the Questionnaire Sections (shallower analysis).

We then chose, for each CP, one OU/ward, and we report the results of every item and Questionnaire Section, obtaining, for each of the four chosen OU/wards, two different performance indexes, one by inserting in the NGSA the priority scores of all the items and the other by using only the mean scores of the Questionnaire Sections.

## 3. Results

**Figure 10:**
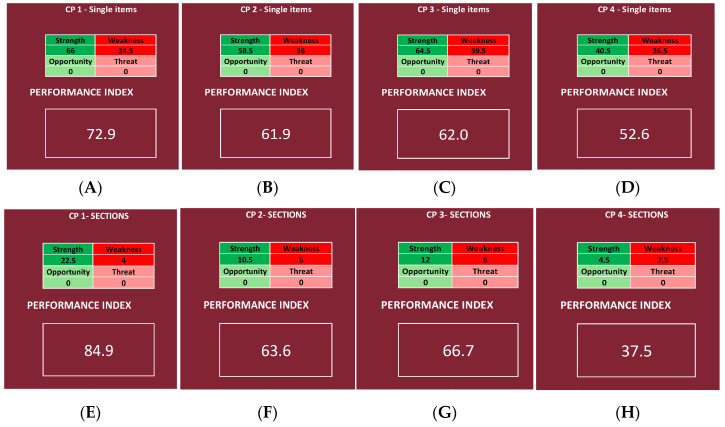
Performance indexes calculated for each CP, using the mean scores of every item of the questionnaire (**A**–**D**) and using the mean scores of every Questionnaire Section (**E**–**H**). For CP1, the deeper analysis is shown in (**A**), and the shallower one is in (**E**); for CP2, the deeper analysis is shown in (**B**), and the shallower one is in (**F**); for CP3, the deeper analysis is shown in (**C**), and the shallower one is in (**G**); lastly, for CP4, the deeper analysis is shown in (**D**), and the shallower one is in (**H**).

**Figure 11:**
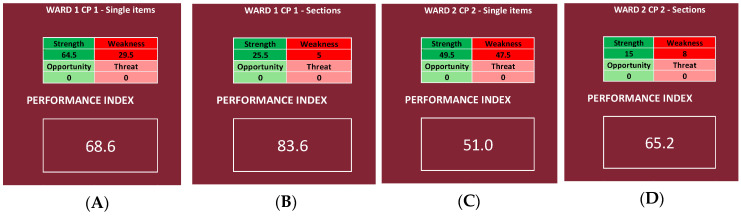

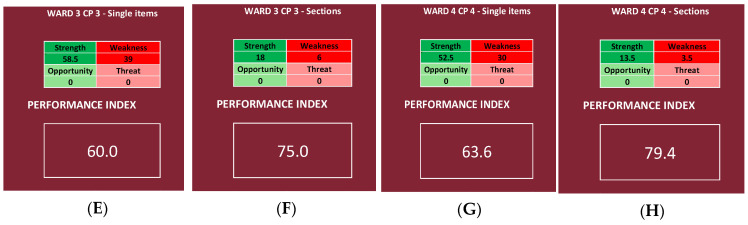
Performance indexes calculated for one randomly chosen OU/ward for each CP, using the mean scores of every item of the questionnaire (**A**,**C**,**E**,**G**) and using the mean scores of every Questionnaire Section (**B**,**D**,**F**,**H**). For Ward 1 of CP1, the deeper analysis is shown in (**A**) and the shallower one in (**B**); for Ward 2 of CP2, the deeper analysis is shown in (**C**) and the shallower one in (**D**); for Ward 3 of CP3, the deeper analysis is shown in (**E**) and the shallower one in (**F**); lastly, for Ward 4 of CP4, the deeper analysis is shown in (**G**) and the shallower one in (**H**).

The results regarding all four CPs are shown in Figure 10, while the results regarding each of the four chosen OU/wards are shown in Figure 11.

The performance indexes obtained with the NGSA were distributed in ascending order to generate two curves, one regarding the items and one regarding the Questionnaire Sections.

The curve regarding the items has a mean of 62.83% and a standard deviation of 0.28, whereas the curve regarding the Questionnaire Sections has a mean of 69.61% and a standard deviation of 0.38.

It was therefore possible to establish that the index is as follows:

- For the items:
  ○ Null if below <52.79% (tenth percentile);
  ○ Low if between 52.80% and 57.55% (between the tenth percentile and the first quartile);
  ○ Good if between 57.56% and 62.83% (between the first and the second quartile);
  ○ High if between 62.84% and 68.12% (between the second and the third quartile);
  ○ Very high if between 68.13% and 100% (between the third and the fourth quartile).
- For the Questionnaire Sections:
  ○ Null if below <51.20% (tenth percentile);
  ○ Low if between 51.21% and 59.92% (between the tenth percentile and the first quartile);
  ○ Good if between 59.93% and 69.61% (between the first and the second quartile);
  ○ High if between 69.62% and 79.30% (between the second and the third quartile);
  ○ Very high if between 79.31% and 100% (between the third and the fourth quartile).

The range reached by each calculated performance index and whether it refers to the items or to the Questionnaire Sections are summarized in Table 3.

### 3.1. All CPs

Looking at Figure 9, it is possible to notice that the index is “very high” in the deeper analysis and “high” in the shallower one, as it is 72.9% (Figure 9A) and 70.6% (Figure 9B), respectively.

**Figure 12:**
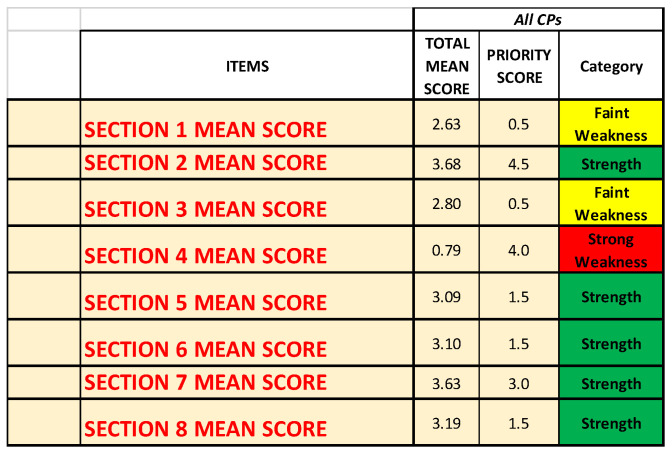
Summary of the results regarding all the Questionnaire Sections of all the CPs together.

**Figure 13:**
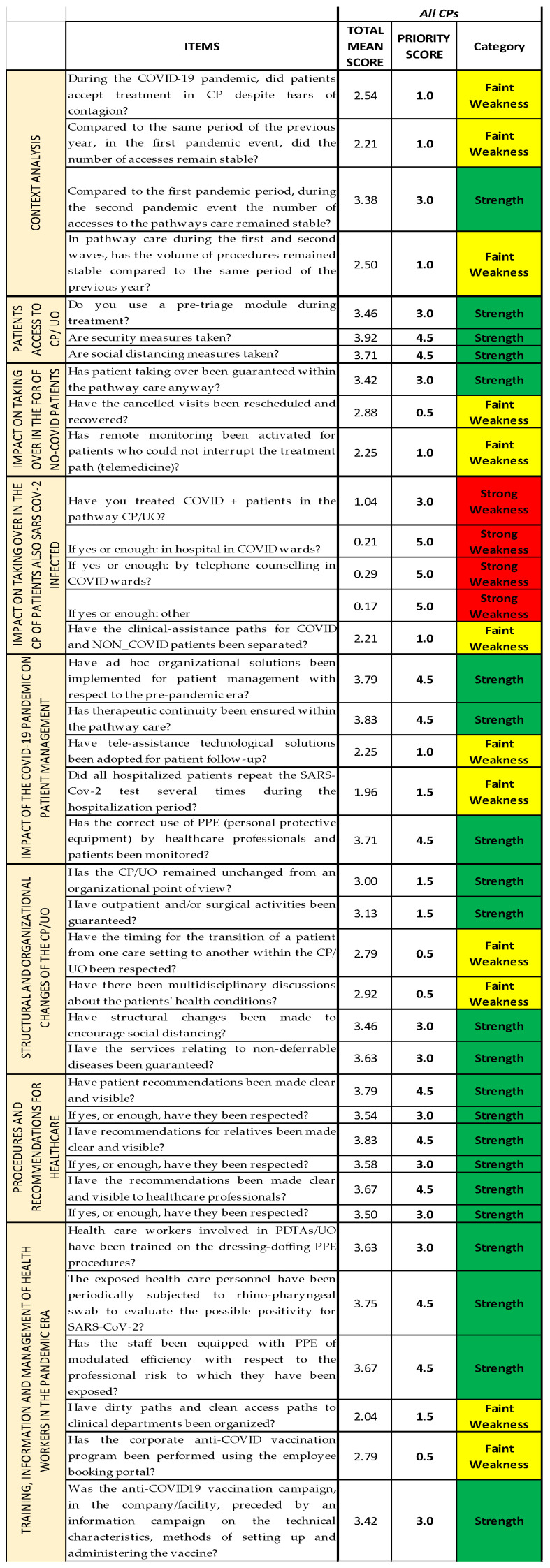
Summary of the results regarding all the items of all the CPs together.

Figure 12 and Figure 13 report the mean scores obtained with the survey, the priority scores inserted into the NGSA, and the category.

Both figures (Figure 12 and Figure 13) show that Questionnaire Section 4 (“Impact on taking over in the CP of patients also SARS-CoV-2 infected”) is the one that is identified as strong weakness.

Figure 12 also highlights how Questionnaire Section 1 (“Context Analysis”) and Questionnaire Section 3 (“Impact on the treatment of non-COVID patients in the CPs”) are both identified as faint weaknesses.

### 3.2. Single CPs

**Figure 14:**
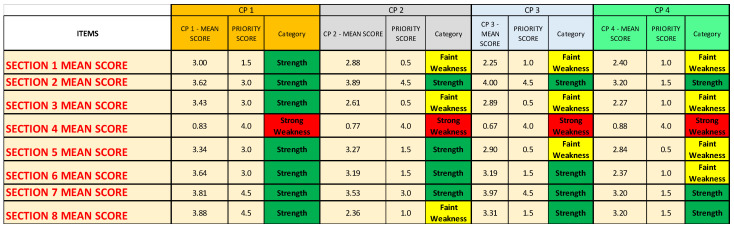
Summary of the results regarding all the Questionnaire Sections of the four analyzed CPs.

**Figure 15:**
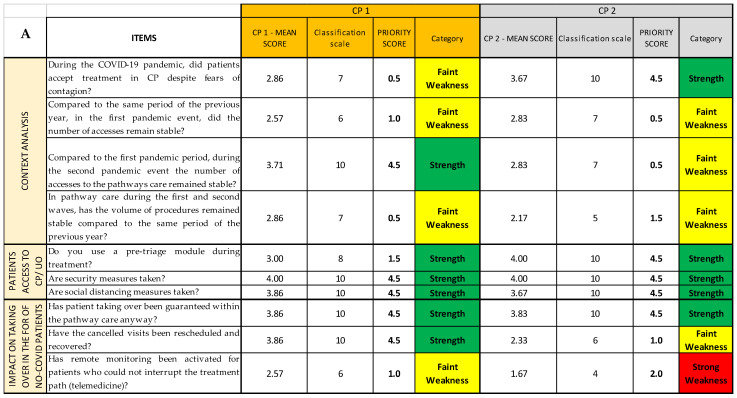

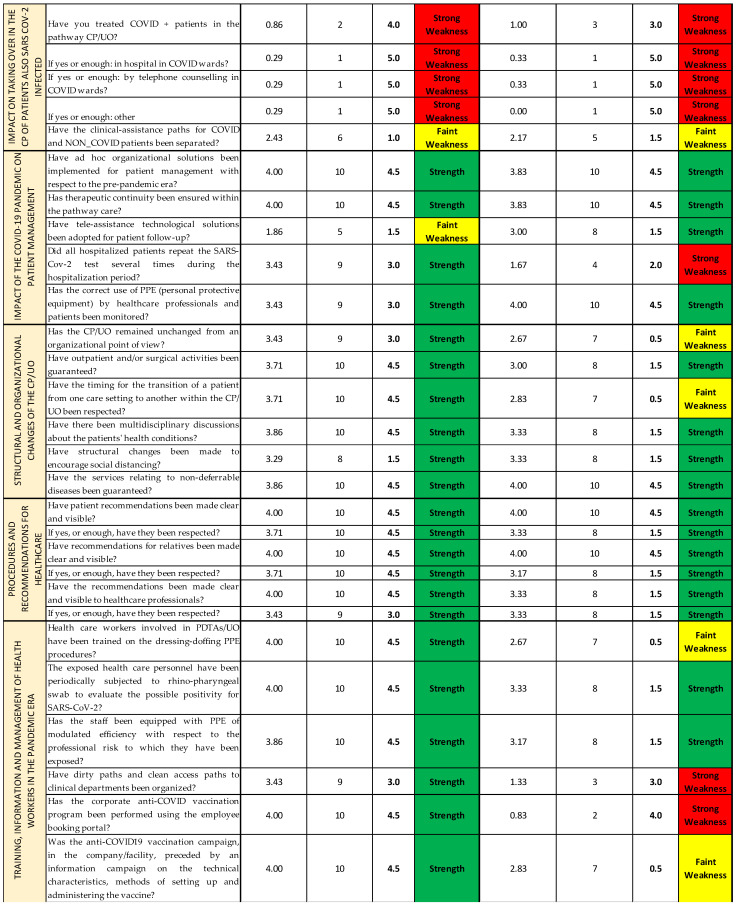

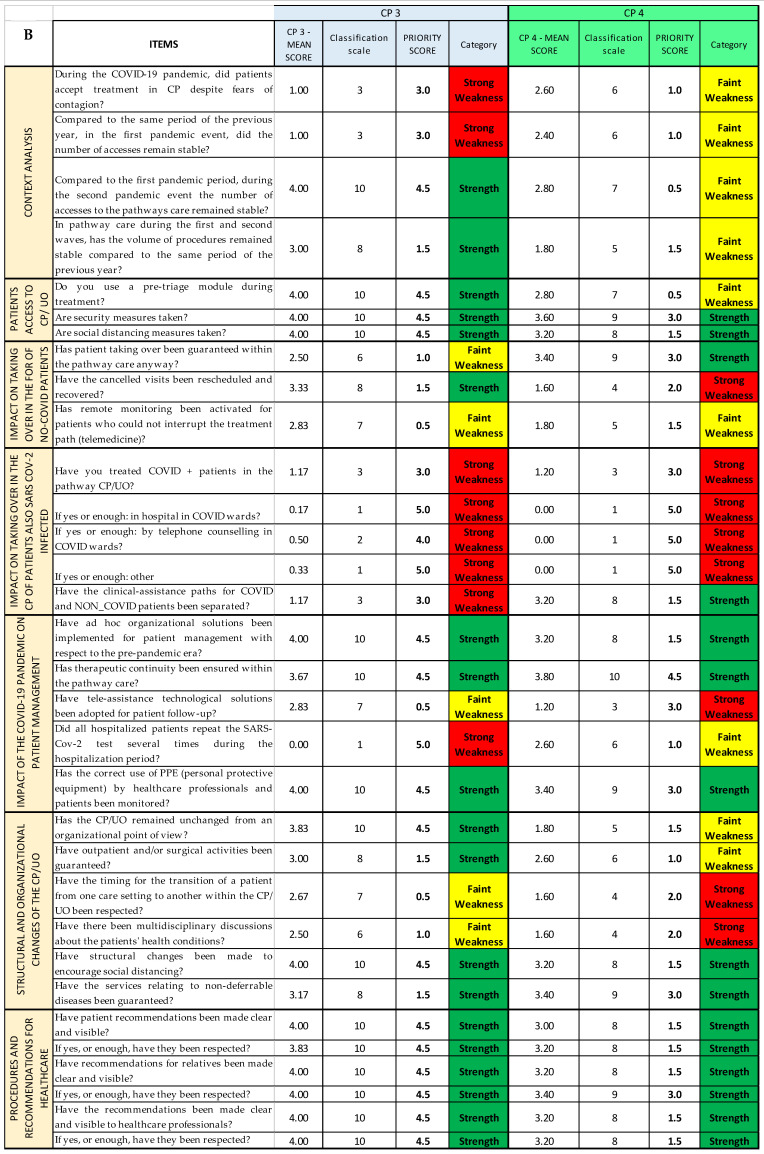

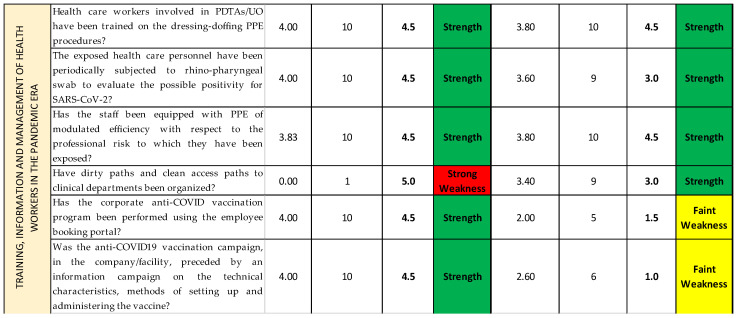
Summary of the results regarding all the items of the four analyzed CPs. (**A**) Results of CP1 and CP2. (**B**) Results of CP3 and CP4.

The results are shown in Figure 10, while Figure 14 and Figure 15 report the mean scores obtained with the survey, the priority scores inserted into the NGSA, and the category.

For CP1 (hereditary breast–ovarian cancers CP), an index of 72.9% was calculated in the deeper analysis (Figure 10A), and 84.9% in the shallower one (Figure 10E), reaching a “very high” range in both cases. For CP2 (autism spectrum disorders CP), 61.9% was calculated in the deeper analysis (Figure 10B), and 63.6% in the shallower one (Figure 10F), reaching a “good” range in both cases. For CP3 (diabetes CP) 62.0% was calculated in the deeper analysis (Figure 10C), and 66.7% in the shallower one (Figure 10G), reaching a “good” range in both cases. Lastly, for CP4 (heart failure CP), 52.6% was calculated in the deeper analysis (Figure 10D), and 37.5% in the shallower one (Figure 10H), reaching a “null” range in both cases.

From the Figures (especially Figure 14), it is possible to notice the following:

- For all four analyzed CPs, Questionnaire Section 4 (“Impact on the treatment of patients also SARS-CoV-2 infected in the CPs”) is identified as a strong weakness;
- Both Questionnaire Section 1 (“Context analysis”) and Questionnaire Section 3 (“Impact on the treatment of non-COVID patients in the CPs”) are identified as a faint weakness for CP2, CP3, and CP4;
- Questionnaire Section 5 (“Impact of the COVID-19 pandemic on patient management”) is identified as a faint weakness for CP3 and CP4;
- Questionnaire Section 6 (“Structural and organizational changes of the CP/OU”) is identified as a faint weakness only for CP4;
- Questionnaire Section 8 (“Training, information, and management of health workers in the pandemic era”) is identified as a faint weakness only for CP2.

### 3.3. Single Wards

For Ward 1 of CP1 (corresponding to OUC Medical Genetics), the deeper analysis (Figure 11A) registers a performance index of 68.6%, whereas the shallower one (Figure 11B) registers an index of 83.6%, reaching a “very high” range in both cases.

For Ward 2 of CP2 (corresponding to OUC Mental Health Center H1–H3), the deeper analysis (Figure 11C) registers a performance index of 51.0%, whereas the shallower one (Figure 11D) registers an index of 65.2%, reaching a “null” and a “good” range respectively.

For Ward 3 of CP3 (corresponding to OUS Primary Care Districts C and D), the deeper analysis (Figure 11E) registers a performance index of 60.0%, whereas the shallower one (Figure 11F) registers an index of 75.0%, reaching a “good” and a “high” range, respectively.

For Ward 4 of CP4 (corresponding to OUD Shock and Trauma), the deeper analysis (Figure 11G) registers a performance index of 63.6%, whereas the shallower one (Figure 11H) registers an index of 79.4%, reaching a “high” and a “very high” range, respectively.

**Figure 16:**
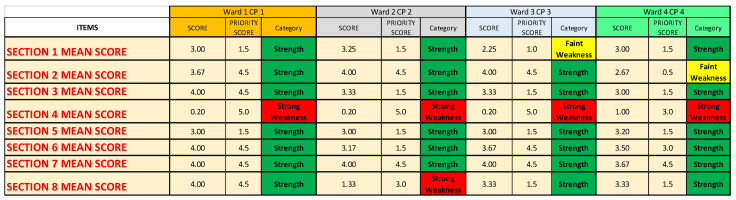
Summary of the results regarding all the Questionnaire Sections of the four wards analyzed, one for each CP.

**Figure 17:**
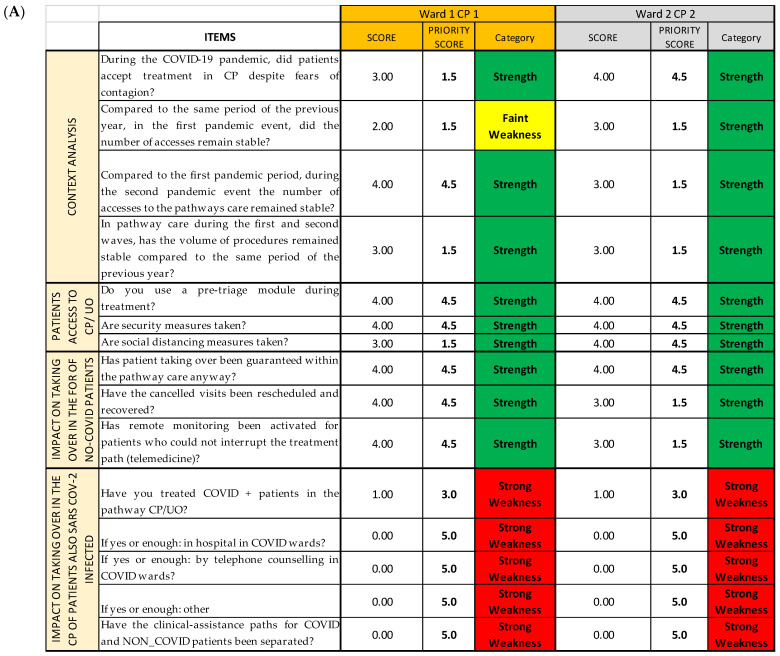

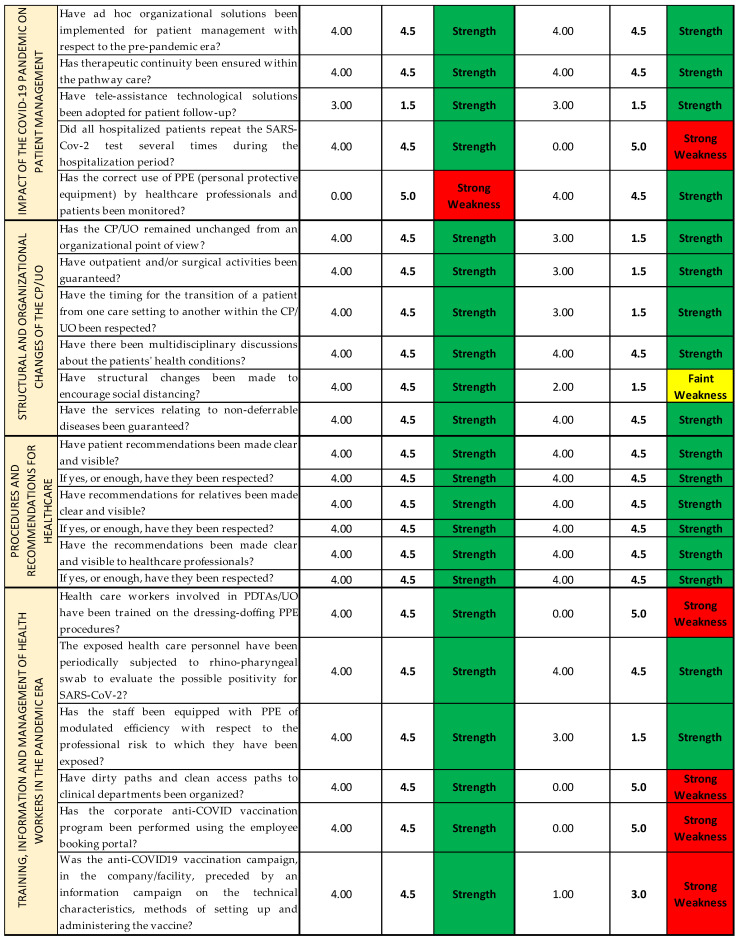

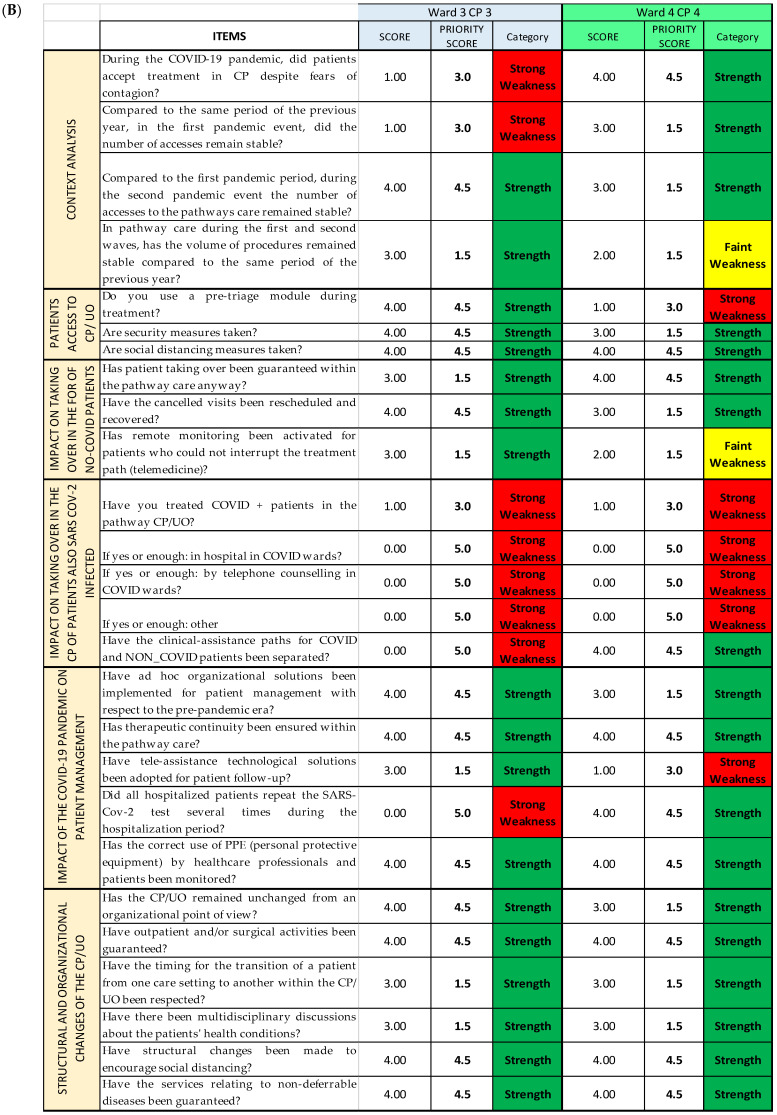

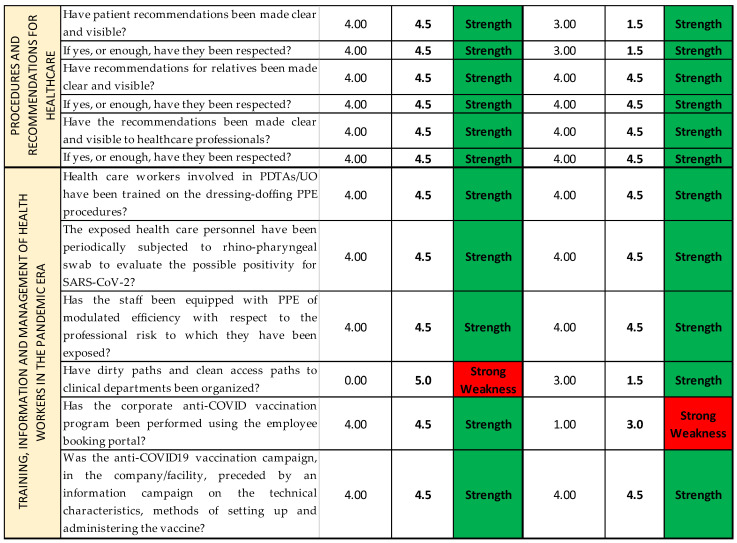
Summary of the results regarding all the items of the four wards analyzed, one for each CP. (**A**) Results of CP1 and CP2. (**B**) Results of CP3 and CP4.

Figure 16 and Figure 17 report the mean scores obtained with the survey, the priority scores inserted into the NGSA, and the category. For all four wards analyzed, Questionnaire Section 4 (“Impact on the treatment of patients also SARS-CoV-2 infected in the CPs”) is identified as a strong weakness, and, for Ward 2 CP2, also Questionnaire Section 8 (“Training, information and management of health workers in the pandemic era”) is identified as a strong weakness.

For Ward 3 CP3 and for Ward 4 CP4, there is also a faint weakness in both Questionnaire Section 1 (“Context analysis”) and Questionnaire Section 2 (“Patients access to care pathways/operational units”), respectively.

## 4. Discussion

The main idea behind this work was to create a methodology for measuring the results of a survey used as an organizational analysis tool in the context of public health policy and to apply it to a previously performed survey regarding the adaptation of pathology-specific care pathways during the first two waves of the COVID-19 pandemic [14]. For this purpose, the Streetlight PRIority Swot system was constructed, allowing us to achieve two main results: establish which sectors of the analyzed organization should be addressed first in the strategic planning of the improvement actions (when deemed necessary to achieve an adequate level of care); and calculate a performance index, through the introduction of the NGSWOT Analysis, that allows us to establish the level of quality of the service offered, as it generates a percentage regarding the survey results as a whole. With the application of the SPRIS on the survey, it was possible to make a direct comparison between the four investigated CPs. For example, when referring to single items, it is possible to find that CP1 fared better than all the others (having a performance index of 72.9%), whereas CP4 fared so poorly that it reaches a “null” performance index (52.6%). Therefore, we can conclude that, regarding CP4, continuity of care was not properly guaranteed. This is a common problem reported in the literature, as the response to the COVID-19 pandemic was the focus on hospital care to prevent the health system from being overburdened during the “state of emergency”, overlooking the importance of primary care in guaranteeing the continuity of care, whilst also ensuring the same therapeutic and diagnostic quality [16,17], reducing outpatient visits number, having multidisciplinary meetings between physicians, and not increasing the work of healthcare workers directly involved in facing the emergency [17]. These findings confirm those of the previous study regarding the COVID survey, as, taking into consideration the total score of each item reported in the previous study, CP1 had the highest score, and CP4 had the lowest [14]. This is confirmed by the literature, as it has been reported that the continuity of oncological care [18,19,20] and all cardiology services (e.g., outpatient clinics, community services, and cardiac rehabilitation) sustained significant reductions [21]. It should be noted that, also in the pre-pandemic era, many studies have sought to identify predictive factors of hospitalization and death and have been based on data obtained from hospital admissions or hospital emergency departments. Instead, the sample selection based on primary care will ensure the inclusion of patients with a wide range of severity, thus improving the risk evaluation [22].

It was also possible to compare different UO/wards, both inside the same CP and between different CPs. Looking at the results, in fact, it can be said that, in the analyzed UO/wards, Ward 1 of CP1 has the best performance index, and Ward 2 of CP2 has the worst one (Figure 11). This is probably due to the fact that, regarding the continuity of care for patients affected by both DSA and SARS-CoV-2, the number of cases was small, and it was not necessary to create separate pathways and/or wards [14], whereas the continuity of oncological care was, in any case, guaranteed thanks to the use of protective devices, pre-triage of patients accessing the hospital, delay of non-urgent visits, and use of telemedicine for patients’ follow-up, in addition to periodical rhino-pharyngeal swabs for SARS-CoV-2 testing in healthcare workers [18,19,20]. It is interesting, however, how the literature reports a gap in the knowledge of palliative care [23] and that a higher education leads to higher chances of survival in some typologies of cancer [24].

The most important element of the SPRIS system is that the first two Excel sheets (*Streetlight color system* and *Priority scores*) erase as much subjectivity as possible, considering that both the classification scales and the priority scores are given following a conversion table. In fact, even by the priority scores, it was already possible to know what areas are faring better than the others, and, therefore, looking at the analysis of the items of every CP, it is noticeable how CP1 has a better level of performance than the others and that CP4 did not manage to properly adapt in front of the pandemic, in contrast to the other three CPs. It is also noticeable how, no matter the type of analysis, Questionnaire Section 4 is always identified as a strong weakness, probably because CPs find their roots in the need for the care management of patients with a specific disease in specific settings, and, therefore, during the pandemic, patients with non-COVID-related illnesses, but SARS-CoV-2 positive, did not follow the specific CP, but were treated within the COVID wards. As a consequence, the SPRIS system identified this as a strong weakness, giving it a high priority score and putting it very high in the decisional matrix.

Moreover, other studies analyzed how their clinical pathways fared in front of the pandemic, considering their key role in reducing the length of stay (LoS) [25] and hospital complications and in improving communication between professionals and safety and quality of care [26], showing varying results. In fact, the first wave of the COVID-19 pandemic has led to a better organization of clinical activities and regular testing among healthcare practitioners, with better chances to grant patients’ protection, underlining the need to develop new protocols for maintaining the good performance of the CPs that are already available [19,21]. This need is highlighted also by the fact that a break in the continuity of care management often leads to decreased general conditions of the patients and healthcare settings [16,27,28,29,30].

Since a different depth of the analysis can be chosen, it is possible to use a superficial analysis in the first place and only apply a deeper analysis to those UO/wards that require it at a later time in order to identify more precisely the issues that have to be addressed. However, it is noticeable how the superficial analysis is less precise and might underestimate or overestimate the problem; in fact, comparing the results of the analysis of the single Questionnaire Sections and those of all the items, a difference can be registered. In this instance, CP1, CP2, and CP3 are overestimated, as the performance indexes calculated using the Questionnaire Sections are higher than the ones obtained using all the items (respectively 12.0, 1.7, and 4.7 points percentage higher, going from 84.9%, 63.6%, and 66.7% in the shallower analysis to 72.9%, 61.9%, and 62.0% in the deeper analysis), and CP4 is underestimated, as the performance index is about 15.1 points lower, registering 52.6% in the deeper analysis and 37.5% in the shallower one. However, going from a more superficial analysis to a deeper one, the range is always the same; in fact, CP1 remains in a “very high” range, CP2 and CP3 remain in a “good” range, and CP4 remains in a “null” range (Table 3).

Moreover, when we considered all the CPs together, the index was high in both cases, confirming that, even when using a bigger dataset, the more superficial analysis is less precise, underestimating the quality of the service.

This is confirmed also by the indexes calculated for the single wards, as Ward 1 of CP1 reaches a “very high” range in both cases; Ward 2 of CP2 reaches a “null” and a “good” range, respectively; Ward 3 of CP3 reaches a “good” and a “high” range, respectively; and, lastly, Ward 4 of CP4 reaches a “high” and a “very high” range, respectively.

The decision to create a single percentage that “summarizes” the findings of the survey, represented by the performance index, was taken with the idea of enhancing the availability of comparable data across countries and over time. In fact, in the literature, there is both a great difficulty in comparing different public health policies with each other [5,29,30,31,32] and a gap regarding the systematic surveillance of public health policies adopted by different states across multiple public health arenas and their evolution and impact on health outcomes [33].

In fact, by generating a single indicator of both the quality and the performance of the whole activity, it is possible to avoid an item-per-item comparison between two surveys, and, therefore, only between common indicators. As a consequence, not only it is a survey data analysis method that can be applied to any kind of survey whose aim is to check the quality of an activity or a service, but it also gives the provider the ability to compare services from different settings, even when using surveys with different items.

### 4.1. Limits of the Study

The authors are aware of some limits. Firstly, our data are quite limited, as they refer to the experience in one region and are not extendible to a national level [14]. In fact, the Italian National Health System (SSN) did not approach the pandemic as a united front, [34,35]. Secondly, at the moment, to our knowledge, there are not any studies in the literature that use survey data to generate a comparable index [36]; therefore, it was impossible to make a comparison.

Thirdly, the SPRIS system still lacks a method of standardization, and it did not undergo a validation process. In fact, the classification of the items into the three categories (strengths, faint weaknesses, and strong weaknesses) is arbitrary, as is the assignation of the priority score, even if the authors did their best to make the logic behind the scores as sound as possible. Moreover, the presence of multiple sheets and various formulas might be a barrier for users. Lastly, it is important to keep in mind that this system can be used in countries with universal healthcare coverage, given the different perspectives of private healthcare systems [5]. Moreover, the use of the performance index as the only indicator of quality of care may hide the fact that, even if the service taken into consideration appears to be adequate in comparison to others, it still falls short of what is attainable through the full application of current medical knowledge [4].

Nevertheless, it is our opinion that the SPRIS system is the first step toward managing to pool data regarding health policy in a meta-analysis.

### 4.2. Further Prospective Work

It must be considered that this is a new theme, and for this reason, it is true that it requires a more in-depth analysis, but it can be a starting point. It is our belief that, considering our results and the limits of this study, the SPRIS system should undergo a full validation process, also in order to eliminate any arbitrary elements left in it. In any case, the SPRIS system needs multiple applications on other surveys, both on other CPs and on other health services, in order to validate our findings. Furthermore, multiple applications would allow the scientific community to pool together the data across countries and over time, finally allowing it to compare different public health policies with each other.

### 4.3. Application

The most important application of the SPRIS system is in the evaluation of the efficacy of the improving measures introduced in a service; by calculating the performance index the first time, the questionnaire is distributed (time zero), and then the performance index is recalculated when the questionnaire is handed out once again after implementing the eventual changes. If, on the second time, the performance index is higher than the first time, then the improving actions were correctly implemented.

Moreover, our index can be used in a direct comparison between different services or organization, allowing us to give to the directional board an immediate overview of the quality of the services, and recognizing with just a glance those that need more resources or those that require a deeper analysis in order to identify the issues that have to be addressed during the elaboration of the improvement actions.

## 5. Conclusions

The SPRIS system showed to be an easily constructed tool that satisfies the needs expressed in the literature regarding an objective and precise overview of a service for strategic planning in healthcare without the need to use complex statistical analysis. It allows users to transform both quality and managerial survey data into an intuitive decision-making matrix that not only identifies the weakness of the system but prioritizes them, which is, to our knowledge, the true innovation brought by this system. A great help in the construction of the SPRIS system as an intuitive decision-making matrix has been the use of the Likert scale; that was the first step towards the conversion of qualitative data into quantitative.

## Figures and Tables

**Table 1:** Comparison between the established cutoffs and the sub-intervals. The established cutoffs allow the synoptic reading of the first Excel sheet (streetlight color system), while the sub-intervals allow us to give the classification scale group.

*Established Cutoffs* [1]	*Sub-Intervals*	*Color*	*Category*	*Classification Scale*
Not Acceptable < 1.80	<0.45	Red	Strong Weakness	1
	0.46–0.89	Red	Strong Weakness	2
	0.90–1.35	Red	Strong Weakness	3
	1.36–1.79	Red	Strong Weakness	4
1.80 ≤ Acceptable ≤ 2.98	1.80–2.20	Yellow	Faint Weakness	5
	2.21–2.60	Yellow	Faint Weakness	6
	2.61–2.98	Yellow	Faint Weakness	7
Good ≥ 2.99	2.99–3.33	Green	Strength	8
	3.34–3.66	Green	Strength	9
	3.67–4.00	Green	Strength	10

**Table 2:** Conversion table. Weaknesses have a descending consecutive scoring system, from 5 to 2 for strong weaknesses, and from 1.5 to 0.5 for faint weaknesses, and strengths have an increasing consecutive scoring system from 1.5 to 4.5. The priority score is to be inserted into the NGSA. The category is colored depending on the streetlight color system.

*Classification Scale*	*Priority Score*	*Category*
1	5	Strong Weakness
2	4	Strong Weakness
3	3	Strong Weakness
4	2	Strong Weakness
5	1.5	Faint Weakness
6	1	Faint Weakness
7	0.5	Faint Weakness
8	1.5	Strength
9	3	Strength
10	4.5	Strength

**Table 3:** Summary of the results regarding the performance indexes and their corresponding ranges.

Performance Index	Subject Analyzed	Subject Name in SPRIS	Kind of Data	Range
72.9%	All CPs	All CPs	Items	Very high
70.6%	All CPs	All CPs	Questionnaire Sections	High
72.9%	Hereditary breast-ovarian cancers CP	CP 1	Items	Very high
84.9%	Hereditary breast-ovarian cancers CP	CP 1	Questionnaire Sections	Very high
61.9%	Autism spectrum disorders (DSA) CP	CP 2	Items	Good
63.6%	Autism spectrum disorders (DSA) CP	CP 2	Questionnaire Sections	Good
62.0%	Diabetes CP	CP 3	Items	Good
66.7%	Diabetes CP	CP 3	Questionnaire Sections	Good
52.6%	Heart failure CP	CP 4	Items	Null
37.5%	Heart failure CP	CP 4	Questionnaire Sections	Null
68.6%	UOC Medical Genetics	Ward 1 CP 1	Items	Very high
83.6%	UOC Medical Genetics	Ward 1 CP 1	Questionnaire Sections	Very high
51.0%	UOC Mental Health Center H1-H3	Ward 2 CP 2	Items	Null
65.2%	UOC Mental Health Center H1-H3	Ward 2 CP 2	Questionnaire Sections	Good
60.0%	UOS Primary Care District C and D	Ward 3 CP 3	Items	Good
75.0%	UOS Primary Care District C and D	Ward 3 CP 3	Questionnaire Sections	High
63.6%	UOD Shock and Trauma	Ward 4 CP 4	Items	High
79.4%	UOD Shock and Trauma	Ward 4 CP 4	Questionnaire Sections	Very high

## Data Availability

All relevant data can be found in the Supplementary Materials.

## Supplementary Materials

The following supporting information can be downloaded at: https://www.mdpi.com/article/10.3390/ijerph19137806/s1, Supplementary Material S1: Delphi-Like Method. Supplementary Material S2: COVID Survey and Survey Results. References [37,38,39] are cited in the supplementary materials.
