# Supplementary material for: From Survey Results to a Decision-Making Matrix for Strategic Planning in Healthcare: The Case of Clinical Pathways
Source: Int J Environ Res Public Health. 2022 Jun 25;19(13):7806. doi: 10.3390/ijerph19137806 (PMC9265412; doi:10.3390/ijerph19137806)
Supplement: Supplementary file 1 [file ijerph-19-07806-s001.zip › ijerph-1752712-supplementary.pdf]

International Journal of
Environmental Research and Public Health

MDPI

Supplementary Material S1: Delphi-Like Method

In the last sheet of the SPRIS system, the results obtained from a survey analyzing the organizational and managerial responses adopted in pathology-specific clinical pathways (CPs) during the first two waves of the COVID-19 pandemic [1] are included in a newly conceived SWOT Analysis (in the form of their respective priority scores), thus allowing us to establish which sectors of the organization should be addressed first in the strategic planning. The aim of the NGSA is to help develop effective public health policies; therefore, the opportunities and threats external to the system that can improve or slow down the company's projects cannot be ignored. If the survey includes elements that analyze opportunities and threats, these items are included in the NGSA, along with their relative priority scores. However, the assigned priority scores must be modified depending on the probability that the event will occur. However, what is the probability that the event will occur? Moreover, who can assign this probability?

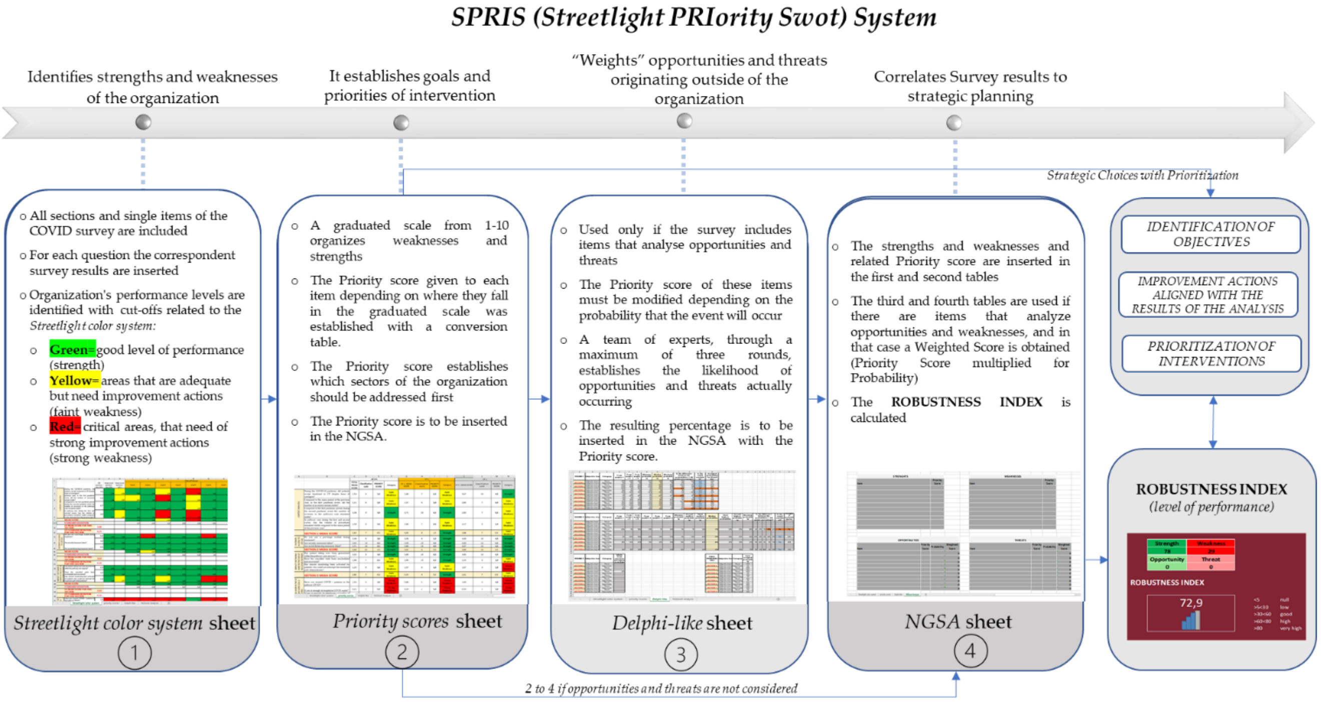

Figure S1. Graphic representation of the construction of the Excel sheets of SPRIS and the meaning of each one of them.

We decided to use a Delphi-like method (Figure S1) in which a team of experts assigns a probability to each opportunity and threat; this system was chosen both because it has been argued that, to achieve our goal, the evidence used must include a wide range of influences [2], and because it was the only possible method to somewhat "standardize" results that, more often than not, refer to area-specific problems. In fact, this method allows us to reduce the subjective component in the assignment of probability, as this was deemed necessary for the correct analysis. Generally speaking, the Delphi method is a

research technique that allows users to regulate and keep track of the communication between a group of experts in order to obtain their opinion in a systematic way and to coagulate subjective judgments; after the construction of a team of experts, the operation is based on the repetition in succession of different "rounds" of interviews or questionnaires on questions and problems until an opinion shared by the entire group is reached [3]. The Delphi techniques are considered highly relevant in health science studies, in both medical/natural science and behavioral/social science disciplines, as the findings of Delphi techniques are often used to draft guidelines or white papers that act as the basis for carrying out and evaluating studies or publications [4].

Even if numerous different variants of Delphi techniques have been developed over the last few years, one of the common objectives is improving predictions of possible future circumstances [4], which is exactly what the Delphi-like method of the SPRIS system is about. However, it is important to keep in mind that, as all experts will have views shaped to some extent by their own interests and experience, bias must be accepted as the rule rather than the exception [1,3]; in order to avoid these unconscious biases that invalidate the results, it is necessary to ensure that there is a good balance in terms of the type of institutions and organizations from which experts are sought. In fact, experts from other disciplines, not necessarily scientific but always according to the criterion of expertise and all classifiable as "local authorities", should also be invited to contribute in order to ensure that the evidence is subjected to a sufficiently questioning review from a wide-ranging set of viewpoints [2]; moreover, the cognitive diversity in an expert group can support innovative and creative discussion processes, and therefore it is just as important for forming a judgment as individual abilities and expertise [4]. In other words, even if a member of the team consistently employs more rigid standards than another or interprets predetermined standards more strictly [5], the application of the Delphi-like system ensures that the probability used to calculate the weighted score falls into the correct dimensional order. The inclusion of local experts is a key point, especially if taking into consideration that one of the main problems of EBI is that organizations, when applying a policy used by others, often make changes to adapt the program to the needs of their community and the capacity of their organization. Such changes can be, for instance, adding, lengthening, shortening, substituting, removing, or re-ordering program components; integrating another approach or intervention; or loosening the structure of intervention [6], and that must be factored in when evaluating eventual problems of the service and whether or not improving actions are required. The fact that EBI adaptations should be documented so that their collective results can be evaluated and their impact be better understood has been underlined [6], and it is our belief that the robustness index calculated with the SPRIS system would be a valid tool when comparing the effectiveness of EBIs that have applied any adaptation, also because qualitative research is the one usually conducted when describing key reasons, types of changes, adaptation processes, and outcomes.

As for the numbers of experts needed, it was decided to put the limit of at least six (three for Round One and three for Round Two), because, in the literature, it is reported

that the average number of experts included in a Delphi technique is usually in the low-to-medium double-digit range (for example, 17 experts or 40 experts), from a minimum of three to a maximum of 731 experts, whereas the most common number of rounds reported in the literature is two or three rounds [4]—hence why we established three rounds. Moving to the selection criteria for the experts, even if the most commons are based on organizational or institutional affiliation, recommendation by third parties, the experience of the experts (measured in years), academic title, or number of publications, in order to guarantee the least amount of bias possible, we suggest that random selection of the experts is used, even if it is rarely reported in the literature [4].

1. Subjective items: opportunities and threats

The role of subjective items is to investigate opportunities and threats external to the organization. Since they are subjective items, their priority scores cannot be used as they are, but they must be weighted; that is, the probability of the event occurring must be considered. Therefore, it will be necessary to use a weighted score, which is calculated by multiplying the priority score of that item for the probability that the event occurs. The probability, being dependent on local factors and therefore hardly found through a review of the literature, is to be found by using a Delphi-like method.

The division between opportunities and threats must be performed at the start of the process, during the designing of the survey questionnaire, so that the authors formulate, when possible, an equal number of questions for each group; it is important to remember that these questions must have the same scoring system of the other questions, as the results need to be uniform. Otherwise, the items in question will not have a score, and it will not be possible to insert them into the NGSA, thus creating a bias in the final result. In fact, the NGSA uses the sum of both the priority scores (strength and weakness) and the weighted scores (opportunities and threats) to calculate a robustness index.

Therefore, these subjective items will be included in the first two Excel sheets with the rest of the data, but, in the *Priority scores* sheet of SPRIS, they will have as category "opportunity" or "threat" instead of strength, strong weakness, or faint weakness.

To this purpose, had we added in our questionnaire at least two items in every section about what the referent thought about the pandemic or the clinical pathways was, we would have categorized the items between opportunities and threats in the survey construction phase (possibly one threat and one opportunity per section), and the possible answers would have been the same as the other items ("yes", "enough", "not enough", "not at all", and "not applicable", where "yes" is equal to 4, "enough" to 3, "not enough" to 2, "not at all" to 1, and "not applicable" to 0) [7], and, therefore, it would have been possible to calculate a mean score and a standard deviation in the exact same way of all the other items and sections. In other words, the Likert scale must be applied to both subjective and objective items, so that the means and standard deviations (SDs) of a bipolar four-point Likert scales can be calculated for each question of the survey. The Likert scale is a very common method of attitude measurement in which the respondent is asked to check one of five possible answers, each one associated with a score from 0 to 4; the final results are the sum of the point values for the choices selected [7].

This would have meant detecting no difference in the *Streetlight color system* sheet nor in the assignation of both the classification scale and the priority score in the *Priority scores* sheet; the only difference visible in the *Priority scores* sheet (Figure S2) would have been represented by the category (opportunity and threat instead of strength and weakness) and by the necessity to generate a probability through the Delphi-like method.

Unfortunately, it was not possible to apply the Delphi-like method to the chosen survey, as it was constructed before the authors created the SPRIS system, and, therefore, it does not include subjective items. However, subjective items and the Delphi-like method

are not necessary for the correct application of the SPRIS, as they are simply an additional element whose aim is to give a better view of the context; its use is not compulsory.

A

A	B	C	D	E	F
		All CPs			
	ITEMS	TOTAL MEAN SCORE	Classification scale	PRIORITY SCORE	Category
IMPACT ON TAKING OVER IN THE FOR OF NO-COVID PATIENTS	Has patient taking over been guaranteed within the pathway care anyway?	3,42	9	3,0	Strength
	Have the cancelled visits been rescheduled and recovered?	2,88	7	0,5	Faint Weakness
	Has remote monitoring been activated for patients who could not interrupt the treatment path (telemedicine)?	2,25	6	1,0	Faint Weakness
	Subjective item 5	2,25	6	1,0	Threat
	Subjective item 6	2,25	6	1,0	Opportunity
	SECTION 3 MEAN SCORE	2,80	7	0,5	Faint Weakness

Streetlight color system | priority scores | Delphi-like | NGSwot Analysis

B

ITEMS	*All CPs* TOTAL MEAN SCOR	Classification scale	PRIORITY SCORE	Category
Subjective item 1	2,50	6	1,0	Threat
Subjective item 2	2,50	6	1,0	Opportunity
Subjective item 3	3,71	10	4,5	Threat
Subjective item 4	3,71	10	4,5	Opportunity
Subjective item 5	2,25	6	1,0	Threat
Subjective item 6	2,25	6	1,0	Opportunity
Subjective item 7	2,21	6	1,0	Threat
Subjective item 8	2,21	6	1,0	Opportunity
Subjective item 9	3,71	10	4,5	Threat
Subjective item 10	3,71	10	4,5	Opportunity
Subjective item 11	3,63	9	3,0	Threat
Subjective item 12	3,63	9	3,0	Opportunity
Subjective item 13	3,50	9	3,0	Threat
Subjective item 14	3,50	9	3,0	Opportunity
Subjective item 15	3,42	9	3,0	Threat
Subjective item 16	3,42	9	3,0	Opportunity

Figure S2. (**A**) How one of the sections of the questionnaire would appear in the *Priority scores* sheet if there had been at least two subjective items in every section. The scores are exemplificative, as are the categories assigned to the items. (**B**) A filter was activated to show the subjective items only.

2. Delphi-like sheet

The Delphi method uses a team of experts to turn qualitative data into quantitative data [3]. In the COVID survey that we bring as an example, it was not necessary to include this process, as subjective items were not included. However, if a survey includes subjective questions that evaluate the opportunities and threats external to the organization that could improve or worsen its situation, the probabilities that the opportunities and threats occur were established with the Delphi-like method, expressed as a percentage that the event will occur, given by a team of experts.

Applying a Delphi-like method to the results of the subjective items ensures that the probability used to calculate the weighted score falls in the correct dimensional order; in order to avoid that the unconscious bias of the team members that can invalidate the results, it is necessary to have a good balance in terms of the type of institutions and organizations from which experts are sought in order to ensure that the evidence is subjected to a sufficiently questioning review from a wide-ranging set of viewpoints [2].

3. Delphi-like method

Each team member gives their opinion anonymously about the probability of a stated impression asked by the item of the questionnaire being actually true (or about the probability of the event brought into question actually happening), giving a percentage from 0% to 100%, where 0% means that the impression is completely false (or that the event will never happen) and 100% means that the impression is completely true (or that the event will happen for sure). The probability put in the NGSA will be the median of the individual answers, based on the principle that the median is closer to the truth than the single idea of a random expert; this is also demonstrated by the fact that, in general, two groups of equally competent experts are more likely to evidence a similar answer to a set of questions than two individuals would [5]. Obviously, the larger the group, the more correct this principle is [1].

Based on this, at least three sets of answers for each item are necessary to have the probability. Moreover, in the first round (Round One), it is preferable not to have a face-to-face discussion, but to just collect individual answers, as it has been demonstrated that group pressure can distort the final result [5].

Therefore, the method we suggest is to randomly assign each item to at least three of the team members, who will send back an answer each, and the median of each item will be calculated. If between the answers given by the three experts there is a big difference—for instance, if two of them say 1% and the last one says 80%—the process has to be repeated (Round Two), as it would mean that the median is not trustworthy. In this case, then, the same item needs to be assigned to three more experts, and the median will be calculated by using all six answers. However, a discussion with the whole team is deemed necessary if, once again, there is a split of opinion (for example, one of them says 3% and the other two say 95%), and, in that case, a consensus between the whole team must be reached (Round Three).

In any case, in order for the median to be trustworthy, the difference between the answers given by the experts must be no higher than 40 points percentages.

Even if it was not necessary for the analysis of the chosen survey [6], we created the *Delphi-like* sheet in order to show how to correctly apply it. For example, had we added in our questionnaire a subjective item for each section about what the referents thought about the pandemic or their insight about the clinical pathways, categorizing the items between opportunities and threats in the survey construction phase, the *Delphi-like* sheet would have been similar to what is shown in Figure S3.

4. Construction of the Delphi-like sheet

The *Delphi-like* sheet contains four different and distinct tables, one for each round (Figure S3) and a final one with all the Final Probabilities. The first two columns are the same for all the tables, as they contain the sections of the questionnaire and the corresponding items, respectively; only the sections that have subjective items and said items are to be included. For instance, if the questionnaire put all the subjective questions in one section rather than putting a few in every section, then the *Delphi-like* sheet's tables will only contain that single section.

In addition, the third column of every table is the same, as it refers to the category to which the item belongs (threat or opportunity).

Starting from the three rounds' tables, the first one, referring to Round One, has 13 columns; the second, referring to Round Two, has 23 columns; and the third, referring to Round Three, has 4 columns; the last one has only four columns because, being a consensus reached after a meeting of the team as a whole, no calculations nor a reorganization of the probabilities given by the experts are necessary. In fact, the tables for Round One and Round Two, in addition to holding the probabilities given by the experts, reorganize them from the lowest probability to the highest one (columns from "Minimum probability" to "Maximum probability"), as it is necessary to judge whether or not there really is a difference higher than 40 points percentage between the given probabilities. Using any other system, the risk to find a difference higher than 40 points percentage just because the subtraction was made between the two extremities of the scale (minimum probability and maximum probability, ignoring all the others in between) would have been too high. This is because, even if the extremities are very far away from each other, if, between the consecutive probabilities that have been reordered from the lowest to the highest, a difference higher than 40 points percentage is not registered, then the round and the median calculated in it are to be considered valid.

The tables for Round One and Round Two also share the construction of the last two columns, "Is the round concluded?" and "Assigned probability".

The aim of the "Is the round concluded?" column is to double-check whether or not the process can end with that round or if it has to go to the next by reporting "No" and coloring itself orange to signal it is necessary another round, or reporting "Yes" and coloring itself light blue if the calculated median can be inserted into the NGSA. This was possible by using not only the Conditional Formatting, as in the other sheets, but also the Excel composite formula:

=IF((COUNT.IF(interval; "> 40%"));"No";"Yes")

where "interval" changes for each row and includes the cells belonging to the two columns answering the question of whether or not there is a difference higher than 40 points percentage between the three probabilities of the subjective item they are referring to. For instance, in Figure S2, using as an example "subjective item 1", the formula is as follows:

=IF((COUNT.IF(J2:K2; "> 40%"));"No";"Yes")

The "Assigned probability" column, instead, reports the median probabilities that were considered valid for that round, using the following composite formula:

=IF(COUNT.IF("Is the round concluded?" cell; "Yes"); median probability cell; "No")

In Figure S2, also using as an example "subjective item 1", the formula is as follows:

= IF(COUNT.IF (L2; "Yes"); H2; "No")

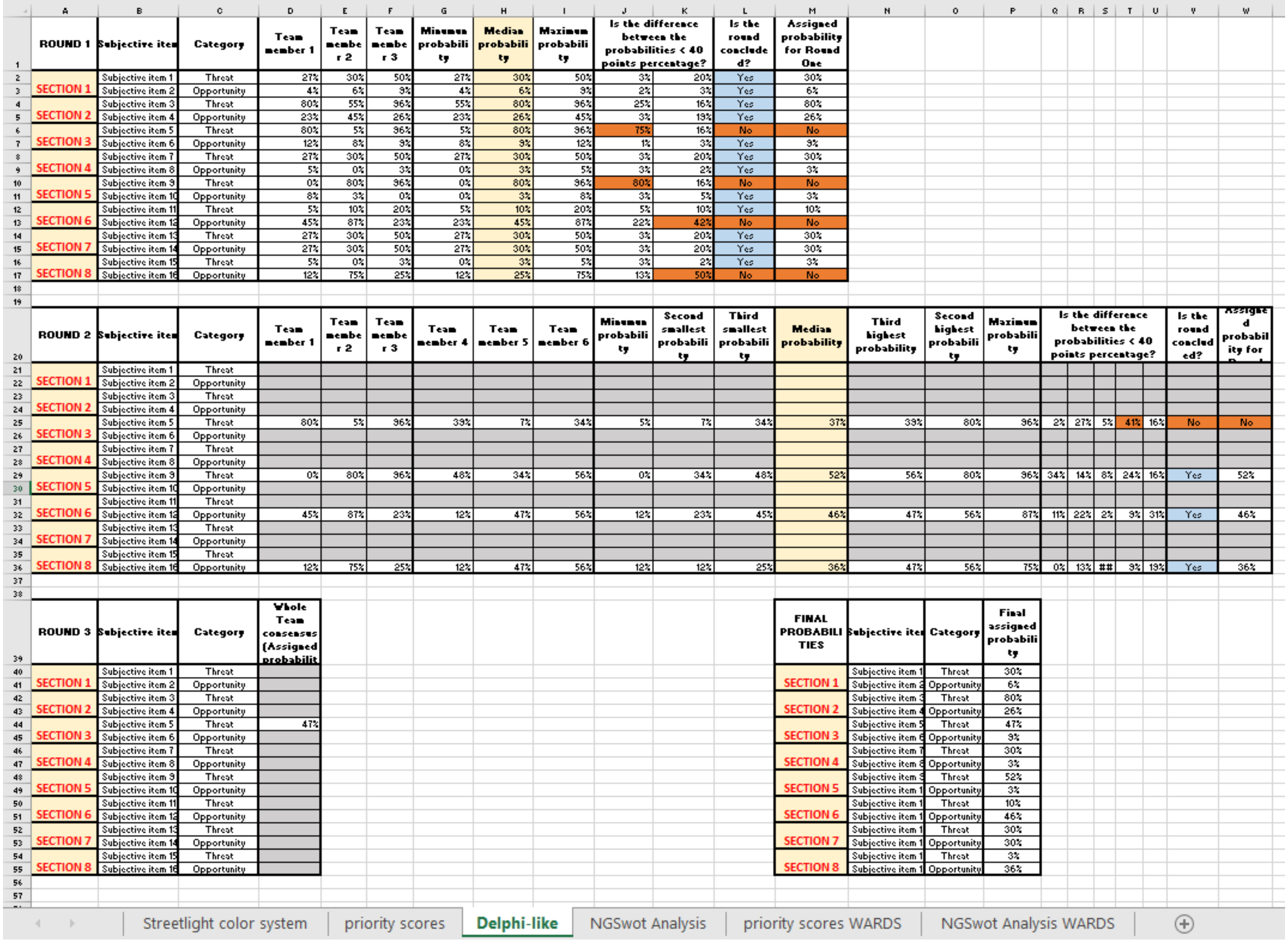

ROUND 1	Subjective item	Category	Team member 1	Team member 2	Team member 3	Minimum probability	Median probability	Maximum probability	Is the difference between the probabilities < 40 points percentage?		Is the round concluded?	Assigned probability for Round One
	Subjective item 1	Threat	27%	30%	50%	27%	30%	50%	3%	20%	Yes	30%
SECTION 1	Subjective item 2	Opportunity	4%	6%	9%	4%	6%	9%	2%	3%	Yes	6%
	Subjective item 3	Threat	80%	55%	96%	55%	80%	96%	25%	16%	Yes	80%
SECTION 2	Subjective item 4	Opportunity	23%	45%	26%	23%	26%	45%	3%	19%	Yes	26%
	Subjective item 5	Threat	80%	5%	96%	5%	80%	96%	75%	16%	No	No
SECTION 3	Subjective item 6	Opportunity	12%	8%	9%	8%	9%	12%	1%	3%	Yes	9%
	Subjective item 7	Threat	27%	30%	50%	27%	30%	50%	3%	20%	Yes	30%
SECTION 4	Subjective item 8	Opportunity	5%	0%	3%	0%	3%	5%	3%	2%	Yes	3%
	Subjective item 9	Threat	0%	80%	96%	0%	80%	96%	80%	16%	No	No
SECTION 5	Subjective item 10	Opportunity	8%	3%	0%	0%	3%	8%	3%	5%	Yes	3%
	Subjective item 11	Threat	5%	10%	20%	5%	10%	20%	5%	10%	Yes	10%
SECTION 6	Subjective item 12	Opportunity	45%	87%	23%	23%	45%	87%	22%	42%	No	No
	Subjective item 13	Threat	27%	30%	50%	27%	30%	50%	3%	20%	Yes	30%
SECTION 7	Subjective item 14	Opportunity	27%	30%	50%	27%	30%	50%	3%	20%	Yes	30%
	Subjective item 15	Threat	5%	0%	3%	0%	3%	5%	3%	2%	Yes	3%
SECTION 8	Subjective item 16	Opportunity	12%	75%	25%	12%	25%	75%	13%	50%	No	No

ROUND 2	Subjective item	Category	Team member 1	Team member 2	Team member 3	Team member 4	Team member 5	Team member 6	Minimum probability	Second smallest probability	Third smallest probability	Median probability	Third highest probability	Second highest probability	Maximum probability	Is the difference between the probabilities < 40 points percentage?					Is the round concluded?	Assigned probability for
	Subjective item 1	Threat																				
SECTION 1	Subjective item 2	Opportunity																				
	Subjective item 3	Threat																				
SECTION 2	Subjective item 4	Opportunity																				
	Subjective item 5	Threat	80%	5%	96%	39%	7%	34%	5%	7%	34%	37%	39%	80%	96%	2%	27%	5%	41%	16%	No	No
SECTION 3	Subjective item 6	Opportunity																				
	Subjective item 7	Threat																				
SECTION 4	Subjective item 8	Opportunity																				
	Subjective item 9	Threat	0%	80%	96%	48%	34%	56%	0%	34%	48%	52%	56%	80%	96%	34%	14%	8%	24%	16%	Yes	52%
SECTION 5	Subjective item 10	Opportunity																				
	Subjective item 11	Threat																				
SECTION 6	Subjective item 12	Opportunity	45%	87%	23%	12%	47%	56%	12%	23%	45%	46%	47%	56%	87%	11%	22%	2%	9%	31%	Yes	46%
	Subjective item 13	Threat																				
SECTION 7	Subjective item 14	Opportunity																				
	Subjective item 15	Threat																				
SECTION 8	Subjective item 16	Opportunity	12%	75%	25%	12%	47%	56%	12%	12%	25%	36%	47%	56%	75%	0%	13%	##	9%	19%	Yes	36%

ROUND 3	Subjective item	Category	Whole Team consensus (Assigned probability
	Subjective item 1	Threat	
SECTION 1	Subjective item 2	Opportunity	
	Subjective item 3	Threat	
SECTION 2	Subjective item 4	Opportunity	
	Subjective item 5	Threat	47%
SECTION 3	Subjective item 6	Opportunity	
	Subjective item 7	Threat	
SECTION 4	Subjective item 8	Opportunity	
	Subjective item 9	Threat	
SECTION 5	Subjective item 10	Opportunity	
	Subjective item 11	Threat	
SECTION 6	Subjective item 12	Opportunity	
	Subjective item 13	Threat	
SECTION 7	Subjective item 14	Opportunity	
	Subjective item 15	Threat	
SECTION 8	Subjective item 16	Opportunity	

FINAL PROBABILITIES	Subjective item	Category	Final assigned probability
	Subjective item 1	Threat	30%
SECTION 1	Subjective item 2	Opportunity	6%
	Subjective item 3	Threat	80%
SECTION 2	Subjective item 4	Opportunity	26%
	Subjective item 5	Threat	47%
SECTION 3	Subjective item 6	Opportunity	9%
	Subjective item 7	Threat	30%
SECTION 4	Subjective item 8	Opportunity	3%
	Subjective item 9	Threat	52%
SECTION 5	Subjective item 1	Opportunity	3%
	Subjective item 1	Threat	10%
SECTION 6	Subjective item 1	Opportunity	46%
	Subjective item 1	Threat	30%
SECTION 7	Subjective item 1	Opportunity	30%
	Subjective item 1	Threat	3%
SECTION 8	Subjective item 1	Opportunity	36%

Figure S3. Photograph of the *Delphi-like* sheet of SPRIS. It shows all four tables, one for each round and the final one; Round One's table has 13 columns, Round Two's table has 23, and Round Three and Final Probabilities have 4. The numbers inserted are exemplificative and not extracted from the questionnaire, as there are no subjective items to which refer to.

4.1. Round One table

The opinions of the first three team members regarding the subjective items are inserted into Round One's table in this Excel sheet in the corresponding columns for each subjective item. The next three columns reorganize the three probabilities, from the lowest to the highest, making the one in the middle the median probability we are actually looking for (salmon pink column in Figure S4). The next two columns answer the question of whether or not there is a difference higher than 40 points percentage between the three probabilities given by the experts. In order to do so, we calculate the difference between the maximum and the median in one column and the median and the minimum in the other, and if one of them is higher than 40%, the corresponding cell colors itself orange. In the same way, the last column tells us if the process can stop here by reporting "No" and coloring itself the same shade of orange to signal that Round Two is necessary, or reporting "Yes" and coloring itself light blue if we can insert into the NSGA the median calculated at this round.

	A	B	C	D	E	F	G	H	I	J	K	L	M
1	ROUND 1	Subjective item	Category	Team member 1	Team member 2	Team member 3	Minumun probability	Median probability	Maximum probability	Is the difference between the probabilities < 40 points percentage?		Is the round concluded?	Assigned probability for Round One
2		Subjective item 1	Threat	27%	30%	50%	27%	30%	50%	3%	20%	Yes	30%
3	SECTION 1	Subjective item 2	Opportunity	4%	6%	9%	4%	6%	9%	2%	3%	Yes	6%
4		Subjective item 3	Threat	80%	55%	96%	55%	80%	96%	25%	16%	Yes	80%
5	SECTION 2	Subjective item 4	Opportunity	23%	45%	26%	23%	26%	45%	3%	19%	Yes	26%
6		Subjective item 5	Threat	80%	5%	96%	5%	80%	96%	75%	16%	No	No
7	SECTION 3	Subjective item 6	Opportunity	12%	8%	9%	8%	9%	12%	1%	3%	Yes	9%
8		Subjective item 7	Threat	27%	30%	50%	27%	30%	50%	3%	20%	Yes	30%
9	SECTION 4	Subjective item 8	Opportunity	5%	0%	3%	0%	3%	5%	3%	2%	Yes	3%
10		Subjective item 9	Threat	0%	80%	96%	0%	80%	96%	80%	16%	No	No
11	SECTION 5	Subjective item 10	Opportunity	8%	3%	0%	0%	3%	8%	3%	5%	Yes	3%
12		Subjective item 11	Threat	5%	10%	20%	5%	10%	20%	5%	10%	Yes	10%
13	SECTION 6	Subjective item 12	Opportunity	45%	87%	23%	23%	45%	87%	22%	42%	No	No
14		Subjective item 13	Threat	27%	30%	50%	27%	30%	50%	3%	20%	Yes	30%
15	SECTION 7	Subjective item 14	Opportunity	27%	30%	50%	27%	30%	50%	3%	20%	Yes	30%
16		Subjective item 15	Threat	5%	0%	3%	0%	3%	5%	3%	2%	Yes	3%
17	SECTION 8	Subjective item 16	Opportunity	12%	75%	25%	12%	25%	75%	13%	50%	No	No

Figure S4. Extraction of the first table constructed in the *Delphi-like* sheet of SPRIS (Round One). It shows the results of Round One. The second to last column tells us if the process can stop here by coloring itself orange to signal Round Two; coloring itself light blue tells us if we can insert into the NSGA the calculated median, which will then appear in the last column. The numbers inserted are exemplificative and not extracted from the questionnaire, as there are no subjective items to which they refer.

4.2. Round Two's table

The table's rows belonging to subjective items that did not need Round Two and whose median can already be inserted into the NGSA are colored in light gray. As for the others, the opinions of the first three team members regarding the subjective items are once again inserted into the corresponding columns for the subjective items (Team Members 1–3), and the next three columns contain in the same way the probabilities given by the next three experts called to give an opinion (Team Members 4–6).

The next seven columns reorganize all six probabilities, from the lowest to the highest, and calculate in the central column the median probability (salmon pink column in Figure S5), which is the number we are looking for to be inserted into the NGSA.

The next five columns answer the question of whether or not there is a difference higher than 40 points percentage between the six probabilities given by the experts. In order to do so, we calculate the difference between the following:

- The maximum and the second highest probability;
- The second highest and the third highest probability;
- The third highest and the third lowest probability;
- The third lowest and the second lowest probability;
- The second lowest and the minimum probability.

In this case, the median does not correspond to one of the probabilities given by the experts, as in Round One, but it is a new number that is calculated by using all six experts' opinions, as the experts' number is an even one; meanwhile, in Round One, it is an odd one. Basically, in this case, after ordering the probabilities in ascending order, there are two middle numbers instead of one, and therefore the median is the mean of these two numbers. Had we added four experts for the Round Two instead of three, then the median would have corresponded to one of the probabilities given, as it would have been an odd number of opinions again. Similar to before, if one of these differences is higher than 40%, the corresponding cell colors itself orange. In the same way, the last column tells us if the process can stop here by reporting "No" and coloring itself the same shade of orange to signal that is necessary to have the whole team to reach a consensus (Round Three), or by reporting "Yes" and coloring itself light blue if we can insert into the NSGA the median calculated at this round, applying the same formula used in Round One's table.

	A	B	C	D	E	F	G	H	I	J	K	L	M	N	O	P	Q	R	S	T	U	V	W
20	ROUND 2	Subjective item	Category	Team member 1	Team member 2	Team member 3	Team member 4	Team member 5	Team member 6	Minumun probability	Second smallest probability	Third smallest probability	Median probability	Third highest probability	Second highest probability	Maximum probability	Is the difference between the probabilities < 40 points percentage?					Is the round concluded?	Assigned probability for Round Two
21		Subjective item 1	Threat																				
22	SECTION 1	Subjective item 2	Opportunity																				
23		Subjective item 3	Threat																				
24	SECTION 2	Subjective item 4	Opportunity																				
25		Subjective item 5	Threat	80%	5%	96%	39%	7%	34%	5%	7%	34%	37%	39%	80%	96%	2%	27%	5%	41%	16%	No	No
26	SECTION 3	Subjective item 6	Opportunity																				
27		Subjective item 7	Threat																				
28	SECTION 4	Subjective item 8	Opportunity																				
29		Subjective item 9	Threat	0%	80%	96%	48%	34%	56%	0%	34%	48%	52%	56%	80%	96%	34%	14%	8%	24%	16%	Yes	52%
30	SECTION 5	Subjective item 10	Opportunity																				
31		Subjective item 11	Threat																				
32	SECTION 6	Subjective item 12	Opportunity	45%	87%	23%	12%	47%	56%	12%	23%	45%	46%	47%	56%	87%	11%	22%	2%	9%	31%	Yes	46%
33		Subjective item 13	Threat																				
34	SECTION 7	Subjective item 14	Opportunity																				
35		Subjective item 15	Threat																				
36	SECTION 8	Subjective item 16	Opportunity	12%	75%	25%	12%	47%	56%	12%	12%	25%	36%	47%	56%	75%	0%	13%	22%	9%	19%	Yes	36%

Figure S5. Extraction of the second table constructed in the *Delphi-like* sheet of SPRIS (Round Two). It shows the results of Round Two. The second to last column tells us if the process can stop here by coloring itself orange to signal Round Three; it colors itself light blue if we can insert into the NSGA the calculated median, which will then appear in the last column. The numbers inserted are exemplificative and not extracted from the questionnaire, as there are no subjective items to which they refer.

4.3. Round Three table

This is the smallest table of the three, as it has only four columns (Figure S6A). As for Round Two's table, the rows referring to subjective items that did not need Round Three and whose median can already be inserted into the NGSA are colored in light gray.

This table reports only the shared opinion about the probability of a stated impression written in the questionnaire being actually true (or about the probability of the event brought into question actually happening) that the experts, after the necessary comparisons, reached.

No calculations are needed in this table, as the team gives one probability only; for the same reason, it is also lacking the last column, as not only the process stops at Round Three in any case, but there is no need for the double check of validity that is guaranteed by the composite formula present in the column.

4.3.1. Final Probabilities table

The last table reports for each subjective item the probability that must be inserted into the NGSA, "fishing" the assigned probability from the last column of every round's table.

In order to do so, it was necessary to create a composite Excel formula that connects all the final columns and copies for each of them the reported number, regardless of which of them contains it; the formula is the following:

=IF(“Is the round concluded” cell =”Yes”;“Assigned probability for Round One” cell ;(IF(“Assigned probability for Round Two” cell=”No”;”Whole Team consensus (Assigned probability for Round Three)” cell; “Assigned probability for Round Two” cell))

For instance, in Figure S6B, using as an example “subjective item 1”, the formula is as follows:

=IF(L2="Yes";M2;(IF(W21="No";D40;W21)))

A

ROUND 3	Subjective item	Category	Whole Team consensus
SECTION 1	Subjective item 1	Threat	
	Subjective item 2	Opportunity	
SECTION 2	Subjective item 3	Threat	
	Subjective item 4	Opportunity	
SECTION 3	Subjective item 5	Threat	47%
	Subjective item 6	Opportunity	
SECTION 4	Subjective item 7	Threat	
	Subjective item 8	Opportunity	
SECTION 5	Subjective item 9	Threat	
	Subjective item 10	Opportunity	
SECTION 6	Subjective item 11	Threat	
	Subjective item 12	Opportunity	
SECTION 7	Subjective item 13	Threat	
	Subjective item 14	Opportunity	
SECTION 8	Subjective item 15	Threat	
	Subjective item 16	Opportunity	

B

P40 =SE(L2="Yes";M2;(SE(W21="No";D40;W21)))

	M	N	O	P	Q	R
39	FINAL PROBABILITIES	Subjective item	Category	Final assigned probability		
40	SECTION 1	Subjective item 1	Threat	30%		
41		Subjective item 2	Opportunity	6%		
42	SECTION 2	Subjective item 3	Threat	80%		
43		Subjective item 4	Opportunity	26%		
44	SECTION 3	Subjective item 5	Threat	47%		
45		Subjective item 6	Opportunity	9%		
46	SECTION 4	Subjective item 7	Threat	30%		
47		Subjective item 8	Opportunity	3%		
48	SECTION 5	Subjective item 9	Threat	52%		
49		Subjective item 10	Opportunity	3%		
50	SECTION 6	Subjective item 11	Threat	10%		
51		Subjective item 12	Opportunity	46%		
52	SECTION 7	Subjective item 13	Threat	30%		
53		Subjective item 14	Opportunity	30%		
54	SECTION 8	Subjective item 15	Threat	3%		
55		Subjective item 16	Opportunity	36%		

Figure S6. Extraction of the third and fourth table constructed in the *Delphi-like* sheet of SPRIS (Round Three and Final Probabilities). (**A**) Results of Round Three. (**B**) Final Probabilities table; the green circle shows the composite Excel formula that connects all the final columns and copies for each of them the reported number applied to subjective item 1. The numbers inserted are exemplificative and not extracted from the questionnaire, as there are no subjective items from which to refer.

References

1. Maria Piane, Lavinia Bianco, Rita Mancini, Paolo Fornelli, Angela Gabriele, Francesco Medici, Claudia Battista, Stefania Greco, Giuseppe Croce, Laura Franceschetti, Christian Napoli, Mario Ronchetti, Paolo Anibaldi, Giorgio Banchieri, "Impact of the COVID-19 Pandemic on Clinical Pathways for Non-SARS-CoV-2 Related Diseases in the Lazio Region, Italy," *International Journal of Environmental Research and Public Health,* vol. 19, no. 2, January 2022.
2. Klein Rudolf, "Evidence and policy: interpreting the Delphic oracle," *Journal of The Royal Society of Medicine,* vol. 98, pp. 429-431, 2003.
3. Norman C. Dalkey, "An experimental study of group opinion: The Delphi method," *Futures,* vol. 1, no. 5, pp. 408-426, 1969.

4. Marlen Niederberger and Julia Spranger, "Delphi Technique in Health Sciences: A Map," *Frontiers in Public Health,* vol. 8, 2020 Sep 22.
5. Avedis Donabedian, "Evaluating the Quality of Medical Care," *The Milbank Quarterly,* vol. 83, no. 4, pp. 691-729, 2005.
6. Cam Escoffery, Erin Lebow-Skelley, Hallie Udelson, Elaine A. Böing, Richard Wood, Maria E. Fernandez and Patricia D. Mullen, "A scoping study of frameworks for adapting public health evidence-based interventions," *TBM,* vol. 9, pp. 1-10, 2019.
7. Drinkwater, B.L. , "A comparison of the direction-of-perception technique with the Likert method in the measurement of attitudes," *J. Soc. Psychol.,* vol. 67, pp. 189-96. , 1965.

Supplementary Material S2: COVID Survey and Survey Results

	COVID SURVEY	Clinical pathways total results
CONTEXT ANALYSIS	During the COVID-19 pandemic, did patients accept treatment despite the fea of contagion?	28% · 32,0% · 12,0% · 20,0% · 8,0%
	Compared to the same period of the previous year, during the first wave of th pandemic, did the number of accesses remain stable?	8% · 40,0% · 12,0% · 40,0%
	Compared with the the first wave of the pandemic, did the number of patient undertaking the care pathways remained stable during the second wave?	64% · 20,0% · 12,0% · 4,0%
	Has the volume of procedures remained stable during first and second wave compared to the same period of the previous year?	12% · 52,0% · 12,0% · 16,0% · 8,0%
PATIENTS ACCESS TO CP/UO	Do you use a pre-triage module during treatment?	88% · 8,0% · 4,0%
	Are security measures taken?	92% · 8,0%
	Are social distancing measures taken?	84% · 16,0%
IMPACT ON THE TREATMENT OF NON-COVID PATIENTS IN THE CLINICAL PATHWAY	Was the treatment to the care pathway garanteed to the patients anyway?	68% · 24,0% · 4,0% · 4,0%
	Have the cancelled visits been rescheduled and recovered?	64% · 16,0% · 0,0% · 20,0%
	Has remote monitoring been activated for patients who could not interrupt th treatment path (telemedicine)?	16% · 40,0% · 16,0% · 8,0% · 20,0%
IMPACT ON THE TREATMENT OF PATIENTS ALSO SARS COV-2 INFECTED IN THE CLINICAL PATHWAY	Have you treated patients affected by COVID-19 within the car pathway/hospital ward?	8,0% · 12,0% · 56,0% · 24,0%
	If yes or enough: in hospital in COVID wards?	4,0% · 12,0% · 84,0%
	If yes or enough: was it in COVID wards with telephone counselling?	8,0% · 12,0% · 80,0%
	Have the clinical-assistance paths for COVID and NON_COVID patients beer separated?	52% · 4,0% · 8,0% · 36,0%
IMPACT OF THE COVID-19 PANDEMIC ON PATIENT MANAGEMENT	Have ad hoc organizational solutions been implemented for patien management with respect to the pre-pandemic era?	92% · 8,0%
	Has therapeutic continuity been ensured within the pathway care?	84% · 16,0%
	Have technological solutions, such as telemedicine, been adopted for patien follow-up?	24% · 32,0% · 20,0% · 4,0% · 20,0%
	Did all hospitalized patients repeat the screening test for SARS-Cov-2 severa times during the hospitalization period?	52% · 4,0% · 44,0%
	Has the correct use of PPE (personal protective equipment) by healthcar professionals and patients been monitored?	96% · 4,0%

	[illegible]	
STRUCTURAL AND ORGANIZATIONAL CHANGES OF THE CPUO	Has the care pathway/hospital ward remained unchanged from ar organizational point of view?	48% · 36,0% · 8,0% · 8,0%
	Have outpatient and/or surgical activities been guaranteed?	60% · 28,0% · 12,0%
	Has the timing of the transition of a patient from one care setting to anothe within the care pathway/hospital ward been respected?	40% · 44,0% · 16,0%
	Have there been multidisciplinary discussions about the patients' healt conditions?	48% · 28,0% · 8,0% · 16,0%
	Have structural changes been made to encourage social distancing?	76% · 16,0% · 4,0% · 4,0%
	Have the services relating to non-deferrable diseases been guaranteed?	88% · 8,0% · 4,0%
PROCEDURES AND RECOMMENDATIONS FOR HEALTHCARE PROFESSIONALS / USERS	Have recommendations for the patients been made clear and visible?	92% · 8,0%
	If yes, or enough, have they been respected?	52% · 48,0%
	Have recommendations for relatives been made clear and visible?	96% · 4,0%
	If yes, or enough, have they been respected?	60% · 36,0% · 4,0%
	Have the recommendations been made clear and visible to healthcar professionals?	92% · 4,0% · 4,0%
	If yes, or enough, have they been respected?	84% · 4,0% · 12,0%
TRAINING, INFORMATION AND MANAGEMENT OF HEALTH WORKERS IN THE PANDEMIC ERA	Have health care workers involved in care pathways/hospital wards bee trained on the dressing-doffing PPE procedures?	88% · 4,0% · 8,0%
	Has the exposed health care personnel been periodically subjected to rhinc pharyngeal swabs to evaluate the possible positivity for SARS-CoV-2?	88% · 8,0% · 4,0%
	Has the staff been equipped with PPE of modulated efficiency with respect t the professional risk to which they have been exposed?	76% · 20,0% · 4,0%
	Have dirty paths and clean access paths to clinical departments bee organized?	44% · 12,0% · 44,0%
	Has the corporate anti-COVID vaccination program been performed using th employee booking portal?	72% · 8,0% · 20,0%
	In the company/facility, was the anti-COVID 19 vaccination campaign precede by an information campaign on the technical characteristics, methods of settin up and administering the vaccine?	88% · 4,0% · 8,0%

Yes · enough · not enough · Not at all · Not applicable

The total score is calculated from the sum of the Likert scale values, where "yes" is equal to 4, "enough" to 3, "not enough" to 2, "not at all" to 1, and "not applicable" to 0.

Context Analysis

1. During the COVID-19 pandemic, did patients accept treatment despite the fear of contagion?

Answer choices	*Responses (number)*	*Responses (score)*
**Yes**	7	7*4=28
**Enough**	8	8*3=24
**Not enough**	2	2*2=4
**Not at all**	5	5*1=5
**Not applicable**	2	2*0=0

Answered	*Total score*	*Mean*	*Variance*	*SD*
24	61	2.54	1.75	1.32

2. Compared to the same period of the previous year, during the first wave of the pandemic, did the number of accesses remain stable?

Answer choices	*Responses (number)*	*Responses (score)*
**Yes**	2	2*4=8
**Enough**	10	10*3=30
**Not enough**	3	3*2=6
**Not at all**	9	9*1=9
**Not applicable**	0	0*0=0

Answered	*Total score*	*Mean*	*Variance*	*SD*
24	53	2.21	1.08	1.04

3. Compared with the first wave of the pandemic, did the number of patients undertaking the care pathways remain stable during the second wave?

Answer choices	*Responses (number)*	*Responses (score)*
**Yes**	15	15*4=60
**Enough**	5	5*3=15
**Not enough**	3	3*2=6
**Not at all**	0	0*1=0
**Not applicable**	1	1*0=0

Answered	*Total score*	*Mean*	*Variance*	*SD*
24	81	3.38	0.98	0.99

4. Has the volume of procedures remained stable during first and second waves compared to the same period of the previous year?

Answer choices	*Responses (number)*	*Responses (score)*
**Yes**	3	3*4=12

**Enough**	13	13*3=39
**Not enough**	3	3*2=6
**Not at all**	3	3*1=3
**Not applicable**	2	2*0=0

Answered	*Total score*	*Mean*	*Variance*	*SD*
24	60	2.50	1.25	1.12

Patients' Access to CP/OU

5. Do you use a pre-triage module during treatment?

Answer choices	*Responses (number)*	*Responses (score)*
**Yes**	20	20*4=80
**Enough**	0	0*3=0
**Not enough**	0	0*2=0
**Not at all**	3	3*1=3
**Not applicable**	1	1*0=0

Answered	*Total score*	*Mean*	*Variance*	*SD*
24	83	3.46	1.50	1.22

6. Are security measures being taken?

Answer choices	*Responses (number)*	*Responses (score)*
**Yes**	22	22*4=88
**Enough**	2	2*3=6
**Not enough**	0	0*2=0
**Not at all**	0	0*1=0
**Not applicable**	0	0*0=0

Answered	*Total score*	*Mean*	*Variance*	*SD*
24	94	3.92	0.08	0.28

7. Are social-distancing measures being taken?

Answer choices	*Responses (number)*	*Responses (score)*
**Yes**	19	19*4=76
**Enough**	4	4*3=12
**Not enough**	0	0*2=0
**Not at all**	1	1*1=1
**Not applicable**	0	0*0=0

Answered	*Total score*	*Mean*	*Variance*	*SD*
24	89	3.71	0.46	0.68

Impact on Access to CPs for Non-COVID Patients

8. Was the start of the treatment within the care pathway guaranteed to the patients anyway?

Answer choices	*Responses (number)*	*Responses (score)*
**Yes**	16	16*4=64
**Enough**	5	5*3=15
**Not enough**	1	1*2=2
**Not at all**	1	1*1=1
**Not applicable**	1	1*0=0

Answered	*Total score*	*Mean*	*Variance*	*SD*
24	82	3.42	1.08	1.04

9. Have the canceled visits been rescheduled and recovered?

Answer choices	*Responses (number)*	*Responses (score)*
**Yes**	14	14*4=56
**Enough**	4	4*3=12
**Not enough**	0	0*2=0
**Not at all**	1	1*1=1
**Not applicable**	5	5*0=0

Answered	*Total score*	*Mean*	*Variance*	*SD*
24	69	2.88	2.61	1.62

10. Has remote monitoring been activated for patients who could not interrupt the treatment (telemedicine)?

Answer choices	*Responses (number)*	*Responses (score)*
**Yes**	4	4*4=16
**Enough**	10	10*3=30
**Not enough**	3	3*2=6
**Not at all**	2	2*1=2
**Not applicable**	5	5*0=0

Answered	*Total score*	*Mean*	*Variance*	*SD*
24	54	2.25	1.94	1.39

Impact on Access to CPs for Patients also Infected by SARS COV-2

11. Have you treated patients affected by COVID-19 within the care pathway/hospital ward?

Answer choices	*Responses (number)*	*Responses (score)*
**Yes**	0	0*4=0
**Enough**	2	2*3=6
**Not enough**	3	3*2=6
**Not at all**	13	13*1=13
**Not applicable**	6	0*0=0

Answered	*Total score*	*Mean*	*Variance*	*SD*
24	25	1.04	0.71	0.84

12. If yes or enough, was it inside the hospital in COVID wards?

Answer choices	Responses (number)	Responses (score)
**Yes**	0	0*4=0
**Enough**	0	0*3=0
**Not enough**	1	1*2=2
**Not at all**	3	3*1=3
**Not applicable**	20	20*0=0

Answered	Total score	Mean	Variance	SD
24	5	0.21	0.25	0.50

13. If yes or enough, was it in COVID wards with telephone counselling?

Answer choices	Responses (number)	Responses (score)
**Yes**	0	0*4=0
**Enough**	0	0*3=0
**Not enough**	2	2*2=4
**Not at all**	3	3*1=3
**Not applicable**	19	19*0=0

Answered	Total score	Mean	Variance	SD
24	7	0.29	0.37	0.61

14. Have the care pathways for COVID and non-COVID patients been separated?

Answer choices	Responses (number)	Responses (score)
**Yes**	12	12*4=48
**Enough**	1	1*3=3
**Not enough**	0	0*2=0
**Not at all**	2	2*1=2
**Not applicable**	9	9*0=0

Answered	Total score	Mean	Variance	SD
24	53	2.21	3.58	1.89

Impact of the COVID-19 Pandemic on Patient Management

15. Have ad hoc organizational solutions been implemented for patient management compared to the pre-pandemic era?

Answer choices	Responses (number)	Responses (score)
**Yes**	21	21*4=84
**Enough**	2	2*3=6
**Not enough**	0	0*2=0
**Not at all**	1	1*1=1
**Not applicable**	0	0*0=0

Answered	Total score	Mean	Variance	SD
24	91	3.79	0.41	0.64

16. Has therapeutic continuity been ensured within the pathway care?

Answer choices	Responses (number)	Responses (score)
**Yes**	20	20*4=80
**Enough**	4	4*3=12
**Not enough**	0	0*2=0
**Not at all**	0	0*1=0
**Not applicable**	0	0*0=0

Answered	Total score	Mean	Variance	SD
24	92	3.83	0.14	0.37

17. Have technological solutions such as telemedicine been adopted for patient follow-up?

Answer choices	Responses (number)	Responses (score)
**Yes**	5	5*4=20
**Enough**	8	8*3=24
**Not enough**	4	4*2=8
**Not at all**	2	2*1=2
**Not applicable**	5	5*0=0

Answered	Total score	Mean	Variance	SD
24	54	2.25	2.02	1.42

18. Did all hospitalized patients repeat the screening test for SARS-Cov-2 several times during the hospitalization period?

Answer choices	Responses (number)	Responses (score)
**Yes**	11	11*4=44
**Enough**	0	0*3=0
**Not enough**	1	1*2=2
**Not at all**	1	1*1=1
**Not applicable**	11	11*0=0

Answered	Total score	Mean	Variance	SD
24	47	1.96	3.71	1.93

19. Has the correct use of PPE (personal protective equipment) by healthcare professionals and patients been monitored?

Answer choices	Responses (number)	Responses (score)
**Yes**	22	22*4=88
**Enough**	0	0*3=0
**Not enough**	0	0*2=0
**Not at all**	1	1*1=1
**Not applicable**	1	1*0=0

Answered	Total score	Mean	Variance	SD
24	89	3.71	0.96	0.98

Structural and Organizational Changes of the CP/OU

20. Has the care pathway/hospital ward remained unchanged from an organizational point of view?

Answer choices	*Responses (number)*	*Responses (score)*
**Yes**	10	10*4=40
**Enough**	9	9*3=27
**Not enough**	2	2*2=4
**Not at all**	1	1*1=1
**Not applicable**	2	2*0=0

Answered	*Total score*	*Mean*	*Variance*	*SD*
24	72	3.00	1.19	1.40

21. Were outpatient and/or surgical activities guaranteed?

Answer choices	*Responses (number)*	*Responses (score)*
**Yes**	14	14*4=56
**Enough**	6	6*3=18
**Not enough**	0	0*2=0
**Not at all**	1	1*1=1
**Not applicable**	3	3*0=0

Answered	*Total score*	*Mean*	*Variance*	*SD*
24	75	3.13	1.86	1.36

22. Has the timing of the transition of a patient from one care setting to another within the care pathway/hospital ward been respected?

Answer choices	*Responses (number)*	*Responses (score)*
**Yes**	9	9*4=36
**Enough**	10	10*3=30
**Not enough**	0	0*2=0
**Not at all**	1	1*1=1
**Not applicable**	4	4*0=0

Answered	*Total score*	*Mean*	*Variance*	*SD*
24	67	2.79	2.00	1.41

23. Have there been multidisciplinary discussions about the patients' health conditions?

Answer choices	*Responses (number)*	*Responses (score)*
**Yes**	12	12*4=48
**Enough**	6	6*3=18
**Not enough**	2	2*2=4
**Not at all**	0	0*1=0
**Not applicable**	4	4*0=0

Answered	*Total score*	*Mean*	*Variance*	*SD*
24	70	2.92	2.08	1.44

24. Have structural changes been made to encourage social distancing?

Answer choices	*Responses (number)*	*Responses (score)*
**Yes**	17	17*4=68
**Enough**	4	4*3=12
**Not enough**	1	1*2=2
**Not at all**	1	1*1=1
**Not applicable**	1	1*0=0

Answered	*Total score*	*Mean*	*Variance*	*SD*
24	83	3.46	1.08	1.04

25. Have the services relating to non-deferrable diseases been guaranteed?

Answer choices	*Responses (number)*	*Responses (score)*
**Yes**	20	20*4=80
**Enough**	2	2*3=6
**Not enough**	0	0*2=0
**Not at all**	1	1*1=1
**Not applicable**	1	1*0=0

Answered	*Total score*	*Mean*	*Variance*	*SD*
24	87	3.63	0.98	0.99

Procedures and Recommendations for Healthcare Professionals/Users

26. Have recommendations for the patients been made clear and visible?

Answer choices	*Responses (number)*	*Responses (score)*
**Yes**	21	21*4=84
**Enough**	2	2*3=6
**Not enough**	0	0*2=0
**Not at all**	1	1*1=1
**Not applicable**	0	0*0=0

Answered	*Total score*	*Mean*	*Variance*	*SD*
24	91	3.79	0.41	0.64

27. If yes or enough, have they been respected?

Answer choices	*Responses (number)*	*Responses (score)*
**Yes**	13	13*4=52
**Enough**	11	11*3=33
**Not enough**	0	0*2=0
**Not at all**	0	0*1=0
**Not applicable**	0	0*0=0

Answered	*Total score*	*Mean*	*Variance*	*SD*
24	85	3.54	0.25	0.50

28. Have recommendations for relatives been made clear and visible?

Answer choices	Responses (number)	Responses (score)
Yes	22	22*4=88
Enough	1	1*3=3
Not enough	0	0*2=0
Not at all	1	1*1=1
Not applicable	0	0*0=0

Answered	Total score	Mean	Variance	SD
24	92	3.83	0.39	0.62

29. If yes or enough, have they been respected?

Answer choices	Responses (number)	Responses (score)
Yes	15	15*4=60
Enough	8	8*3=24
Not enough	1	1*2=2
Not at all	0	0*1=0
Not applicable	0	0*0=0

Answered	Total score	Mean	Variance	SD
24	86	3.58	0.33	0.57

30. Have recommendations for healthcare professionals been made clear and visible?

Answer choices	Responses (number)	Responses (score)
Yes	21	21*4=84
Enough	1	1*3=3
Not enough	0	0*2=0
Not at all	1	1*1=1
Not applicable	1	1*0=0

Answered	Total score	Mean	Variance	SD
24	88	3.67	0.97	0.99

31. If yes or enough, have they been respected?

Answer choices	Responses (number)	Responses (score)
Yes	20	20*4=80
Enough	1	1*3=3
Not enough	0	0*2=0
Not at all	1	1*1=1
Not applicable	2	2*0=0

Answered	Total score	Mean	Variance	SD
24	84	3.50	1.50	1.22

Training, Information, and Management of Health Workers in the Pandemic Era

32. Have healthcare workers involved in care pathways/hospital wards been trained on the dressing–doffing PPE procedures?

Answer choices	*Responses (number)*	*Responses (score)*
**Yes**	21	21*4=84
**Enough**	1	1*3=3
**Not enough**	0	0*2=0
**Not at all**	0	0*1=0
**Not applicable**	2	2*0=0

Answered	*Total score*	*Mean*	*Variance*	*SD*
24	87	3.63	1.23	1.11

33. Has the exposed healthcare personnel been periodically subjected to rhino-pharyngeal swabs to evaluate the possible positivity for SARS-CoV-2?

Answer choices	*Responses (number)*	*Responses (score)*
**Yes**	21	21*4=84
**Enough**	2	2*3=6
**Not enough**	0	0*2=0
**Not at all**	0	0*1=0
**Not applicable**	1	1*0=0

Answered	*Total score*	*Mean*	*Variance*	*SD*
24	90	3.75	0.69	0.83

34. Has the staff been equipped with PPE of modulated efficiency with respect to the professional risk to which they have been exposed?

Answer choices	*Responses (number)*	*Responses (score)*
**Yes**	19	19*4=76
**Enough**	4	4*3=12
**Not enough**	0	0*2=0
**Not at all**	0	0*1=0
**Not applicable**	1	1*0=0

Answered	*Total score*	*Mean*	*Variance*	*SD*
24	88	3.67	0.72	0.85

35. Have dirty paths and clean access paths to clinical departments been organized?

Answer choices	Responses (number)	Responses (score)
**Yes**	10	10*4=40
**Enough**	3	3*3=9
**Not enough**	0	0*2=0
**Not at all**	0	0*1=0
**Not applicable**	11	11*0=0

Answered	Total score	Mean	Variance	SD
24	49	2.04	3.62	1.90

36. Has the corporate anti-COVID vaccination program been performed by using the employee booking portal?

Answer choices	Responses (number)	Responses (score)
**Yes**	16	16*4=64
**Enough**	0	0*3=0
**Not enough**	0	0*2=0
**Not at all**	3	3*1=3
**Not applicable**	5	5*0=0

Answered	Total score	Mean	Variance	SD
24	67	2.79	3.00	1.73

37. In the company/facility, was the anti-COVID19 vaccination campaign preceded by an information campaign on the technical characteristics, namely methods of setting up and administering the vaccine?

Answer choices	Responses (number)	Responses (score)
**Yes**	20	20*4=80
**Enough**	0	0*3=0
**Not enough**	0	0*2=0
**Not at all**	2	2*1=2
**Not applicable**	2	2*0=0

Answered	Total score	Mean	Variance	SD
24	82	3.42	1.74	1.32
